# Worldwide variation in cardiovascular magnetic resonance practice models

**DOI:** 10.1186/s12968-023-00948-7

**Published:** 2023-07-03

**Authors:** Lilia M. Sierra-Galan, Edgar E. S. Estrada-Lopez, Victor A. Ferrari, Subha V. Raman, Vanessa M. Ferreira, Vimaj Raj, Elizabeth Joseph, Jeanette Schulz-Menger, Carmen W. S. Chan, Sylvia S. M. Chen, Yuchen Cheng, Juliano De Lara Fernandez, Masahiro Terashima, Timothy S. E. Albert

**Affiliations:** 1 American British Cowdray Medical Center, Mexico City, Mexico; 2 Association of Scouts of Mexico, Mexico City, Mexico; 3 Hospital of the University of Pennsylvania and Penn Cardiovascular Institute, Cardiovascular Division, Perelman School of Medicine, University of Pennsylvania, Philadelphia, PA USA; 4 Indiana University School of Medicine, Indianapolis, IN USA; 5 Oxford Centre for Clinical Magnetic Resonance Research, University of Oxford, John Radcliffe Hospital, Oxford, UK; 6 Narayana Hrudayalaya Institute of Cardiac Sciences, Hosur Road, Bangalore, India; 7 Christian Medical College, Vellore, Tamil Nadu India; 8 Charité, University Medicine Berlin, ECRC, Helios-Clinics, DZHK-Partner Site Berlin, Berlin, Germany; 9 Queen Mary Hospital, Hong Kong, China; 10 The Northern Hospital, Melbourne, Australia; 11 West China Hospital, Sichuan University, Leshan, Sichuan China; 12 Radiologia Clinica De Campinas, Instituto de Ensino e Pesquisa Jose Michel Kalaf, Campinas, Brazil; 13 CVIC (Cardiovascular Imaging Clinic), Tokyo, Japan; 14 Huntington Hospital, Pasadena, CA USA

**Keywords:** CMR, Cardiovascular magnetic resonance, Survey, Practice models, World

## Abstract

**Introduction:**

The use of cardiovascular magnetic resonance (CMR) for diagnosis and management of a broad range of cardiac and vascular conditions has quickly expanded worldwide. It is essential to understand how CMR is utilized in different regions around the world and the potential practice differences between high-volume and low-volume centers.

**Methods:**

CMR practitioners and developers from around the world were electronically surveyed by the Society for Cardiovascular Magnetic Resonance (SCMR) twice, requesting data from 2017. Both surveys were carefully merged, and the data were curated professionally by a data expert using cross-references in key questions and the specific media access control IP address. According to the United Nations classification, responses were analyzed by region and country and interpreted in the context of practice volumes and demography.

**Results:**

From 70 countries and regions, 1092 individual responses were included. CMR was performed more often in academic (695/1014, 69%) and hospital settings (522/606, 86%), with adult cardiologists being the primary referring providers (680/818, 83%). Evaluation of cardiomyopathy was the top indication in high-volume and low-volume centers (p = 0.06). High-volume centers were significantly more likely to list evaluation of ischemic heart disease (e.g., stress CMR) as a primary indicator compared to low-volume centers (p < 0.001), while viability assessment was more commonly listed as a primary referral reason in low-volume centers (p = 0.001). Both developed and developing countries noted cost and competing technologies as top barriers to CMR growth. Access to scanners was listed as the most common barrier in developed countries (30% of responders), while lack of training (22% of responders) was the most common barrier in developing countries.

**Conclusion:**

This is the most extensive global assessment of CMR practice to date and provides insights from different regions worldwide. We identified CMR as heavily hospital-based, with referral volumes driven primarily by adult cardiology. Indications for CMR utilization varied by center volume. Efforts to improve the adoption and utilization of CMR should include growth beyond the traditional academic, hospital-based location and an emphasis on cardiomyopathy and viability assessment in community centers.

**Supplementary Information:**

The online version contains supplementary material available at 10.1186/s12968-023-00948-7.

## Introduction

Since its inception in the 1970s, nuclear magnetic resonance (later renamed magnetic resonance imaging or MRI) has evolved from a novel imaging modality into the clinical mainstream for routine diagnosis and management of patients. Technological advancement for in vivo MRI of the heart and vasculature in humans has matured over the past 40 years. As a result, virtually all conditions affecting the structure and function of the cardiovascular system—ranging from congenital disorders to ischemic and non-ischemic diseases—can be assessed non-invasively with high precision using modern-day cardiovascular magnetic resonance (CMR) in routine clinical settings.

As CMR is rapidly expanding its clinical application worldwide, it is essential to understand how this technology is being used and who is performing it. This will help develop new CMR programs and inform societal efforts to ensure quality and excellence across centers. Over the years, CMR has accumulated strong evidence for its incorporation into international guidelines and appropriate use criteria by leading international societies, such as the American College of Cardiology (ACC), the American Heart Association (AHA), and the European Society of Cardiology (ESC) [1–18]. However, local CMR practices often vary. This work aimed to electronically survey clinical practitioners and other members of the CMR community from around the world, to better understand how and where CMR is practiced, to characterize variations in training and experience, and to better define barriers and challenges for the future growth of CMR.

## Methods

Two electronic surveys were used to obtain specific information worldwide using Survey Monkey® (https://www.surveymonkey.com). The first survey was emailed to all SCMR members through its email distribution lists, asking them to answer the questions based on their personal, local, and/or regional experience and to forward the survey to their contact lists to reach out to non-SCMR members. This survey aimed to understand better how our members and other practitioners deliver CMR services to their community. The second version of the survey was an expanded version of the previous one based on user feedback. We kept the same format and questions, removed unnecessary ones, and added new queries for additional details in certain areas, such as economics. We also invited industry partners within the MR community to participate in this survey. The complete list of survey questions used is provided in the Additional file 1. The population of each country at the time of surveying was derived from publicly available data from the United Nations [19].

Survey results were obtained and collated by administrators at SCMR headquarters and then distributed to the leaders of the project (LSG and TA) and to a data expert (EESEL) to review and carefully curate the data before merging both to avoid duplicates and errors in responses that only a CMR expert could identify. Our data expert (EESEL) organized, collated, and analyzed the data using the Qlik® SENSE (Qlik Technologies Inc., King of Prussia, Pennsylvania, USA) and Google Data Studio (Google Data Studio Cloud, Mountain View, California, USA) platforms. After careful review and team discussion of the obtained data, we considered the data curation phase concluded. Then, the data expert proceeded to the data integration phase by incorporating two previously curated files, the first with 360 records in 145 fields and the second with 732 records in 104 fields. Next, a field map was created, matching the columns of one survey with equivalent columns of the second survey. Finally, both files were integrated into one single survey result. There were 38 effective duplicate records (37 users had answered both surveys—one of them had responded three times), with duplicate records identified by creating the unique key of each record by using email address + gender + country of origin + age; and by cross-referencing to the IP address of the computer system used to respond. We discarded the first duplicated records we found on each survey and only used the most recent record.

Specific examples of the data curation processes used are described in more detail in the Additional file 1. Statistical analysis was performed in Microsoft Excel® (Microsoft, One Microsoft Way, Redmond, Washington, USA) and Google Data Studio. Continuous variables were reported using mean, standard deviations, and percentages, presented as mean and range for skewed data.

## Results

### Demographics

**Fig. 1:**
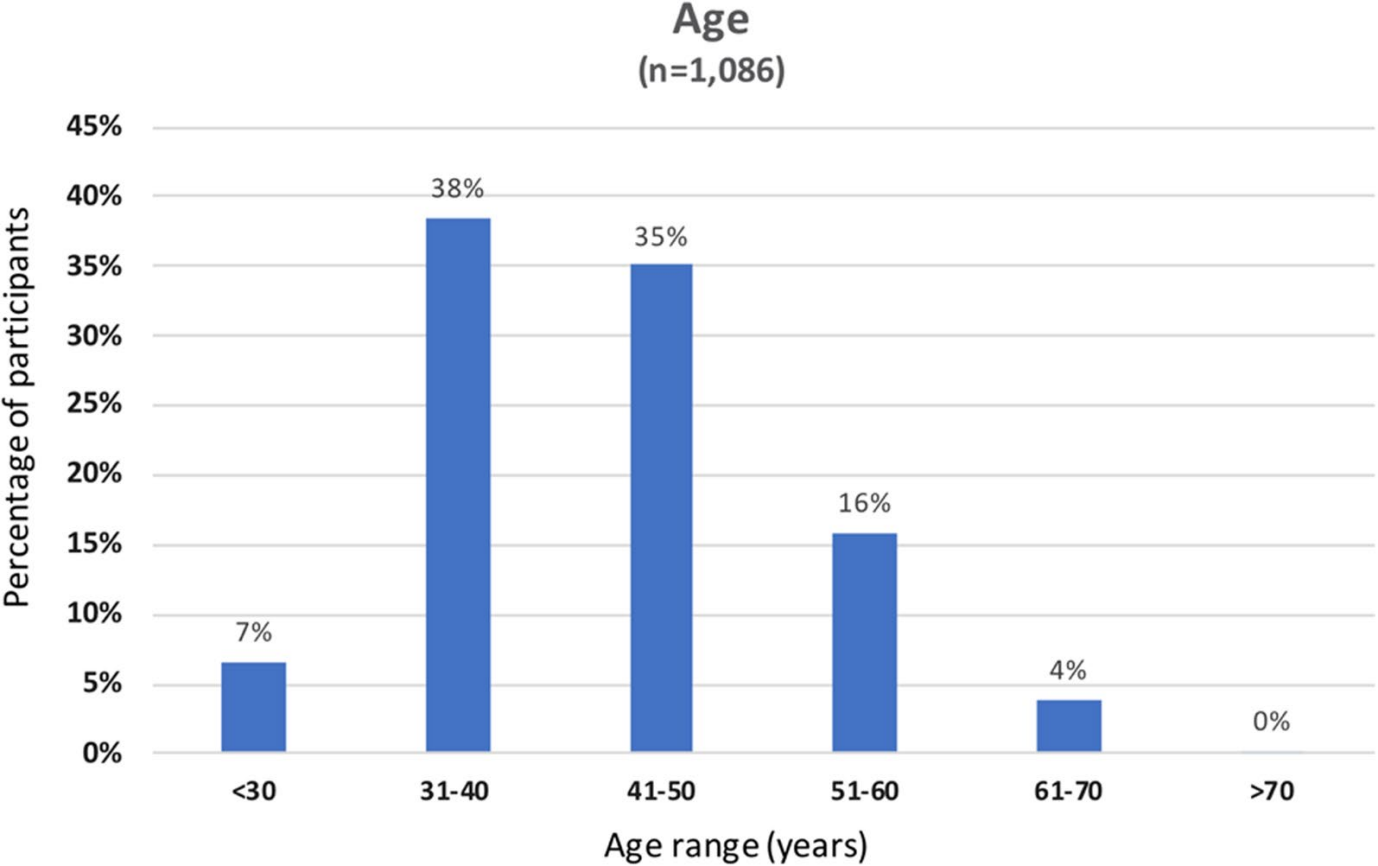
Age distribution

Surveys were returned from 70 different countries with a total of 1092 unique responses from at least 489 unique sites (not all responders provide a location of practice). (See Table 1). The top five countries (United States, United Kingdom, Brazil, Germany, and Japan) provided 58% of the obtained responses. The majority (798/1092, 73%) of the responders were between 31 to 50 years of age (see Fig. 1), with (749/1092, 69)% of the respondents being male and (337/1092, 31%) female. The top three categories of responders practiced adult cardiology (423/884, 48%), adult radiology (193/884, 22%), and pediatric cardiology (96/884, 11%). Additionally, various categories of non-clinical responders were represented, including technologists (50/884, 6%), non-clinical scientists (30/884, 3.4%), and medical industry representatives (21/884, 2.4%). Of the survey responders, (564/1090, 52%) reported being SCMR members, with (363/564, 64%) of those having full membership and the remaining associates, trainees (male (55/564, 9.8% and female (41/564, 7.3%), and technologists (male 9/564, 1.6% and female 27/564, 4.8%) (see Additional file 1).

**Table 1:** Survey response and classification of countries according to the United Nations 2018 economic indicators

Surveyed countries (n = 70)					
Developed	n = 777(%)	No. unique sites surveyed	Developing	n = 284 (%)	No. unique sites surveyed
United States	309 (40)	127	Brazil	85 (30)	47
United Kingdom	112 (14)	37	India	31 (11)	16
Germany	62 (8)	22	China	18 (6)	11
Japan	50 (6)	7	South Africa	17 (6)	9
Canada	42 (5)	23	Malaysia	15 (5)	9
The Netherlands	30 (4)	13	Mexico	15 (5)	3
Italy	22 (3)	15	Egypt	10 (4)	8
Switzerland	22 (3)	12	Singapore	10 (4)	5
Australia	18 (2)	12	Argentina	9 (3)	6
Spain	18 (2)	9	Hong Kong	8 (3)	N/A
Sweden	12 (2)	6	Saudi Arabia	8 (3)	6
Greece	10 (1.3)	5	Thailand	8 (3)	3
France	8 (1.0)	5	Colombia	6 (2)	4
Ireland	7 (0.9)	3	Indonesia	4 (1.4)	4
Norway	7 (0.9)	3	Chile	3 (1.1)	1
Austria	6 (0.8)	3	Kuwait	3 (1.1)	2
Portugal	6 (0.8)	4	Philippines	3 (1.1)	3
Denmark	5 (0.6)	3	South Korea	5 (1.8)	2
Hungary	5 (0.6)	3	Uruguay	3 (1.1)	2
New Zealand	5 (0.6)	3	Algeria	2 (0.7)	1
Czech Republic	4 (0.5)	3	Bangladesh	2 (0.7)	1
Lithuania	4 (0.5)	2	El Salvador	2 (0.7)	1
Romania	4 (0.5)	2	Qatar	2 (0.7)	1
Belgium	3 (0.4)	2	United Arab Emirates	2 (0.7)	1
Finland	2 (0.3)	2			
Andorra	1 (0.1)	1	Ecuador	1 (0.4)	1
Monaco	1 (0.1)	1	Iran	1 (0.4)	1
Poland	1 (0.1)	1	Kazakhstan	1 (0.4)	1
Slovakia	1 (0.1)	1	Lebanon	1 (0.4)	1
*Classification according to the United Nations* *2018 economic indicators*			Mongolia	1 (0.4)	1
*In-transition*	*n = 10*	*No. unique sites surveyed*	Morocco	1 (0.4)	1
Turkey	8 (80)	4	Myanmar	1 (0.4)	1
Georgia	1 (10)	1	Nicaragua	1 (0.4)	1
Russia	1 (10)	1	Oman	1 (0.4)	1
			Pakistan	1 (0.4)	1
			Panama	1 (0.4)	1
			Venezuela	1 (0.4)	1
			Vietnam	1 (0.4)	1

This table shows the number of responders (%) to the survey and the number of unique sites surveyed and classified by developed, in-transition, and developing countries according to countries' United Nations economic classification [22]. Sample size of each category is different since not all responders answered all questions

### Training, experience, and professional activity

Of the responders, 333/923 (36%) reported formal training, and 590/923 (64%) reported no formal training. Most of the responders reported either being CMR staff/faculty 119/304 (39%) or medical directors of their department (137/304, 45%) (see Table 2). When asked about experience level, 285/641 (44%) reported > 10 years of experience with CMR, with 495/1041 (45%) reporting some official CMR certification or verification of competence: SCMR certification in 110/495 (22%), EACVI in 58/495 (12%), SCMR plus EACVI in 51/495 (10%), and a different CMR certification not specified in 276/495 (56%). Only 64/1024 (6%) of responders reported spending > 75% of their time supervising or reading CMR studies, with the majority (522/1024, 51%) spending ≤ 50% of their time on CMR-related activities (data not shown).

**Table 2:** Experience, training, and professional activity

CMR training, experience, and professional activity		
Formal training	n = 923	%
Yes	333	36
No	590	64

Level of expertise	n = 304	%
Fellow	48	16
Staff/Faculty	119	39
Medical director of the department	137	45

Years of experience	n = 641	%
< 1	24	4
1–2	67	10
3–5	99	15
6–10	166	26
> 10	285	44

Official certification/verification of competence	n = 1092	%
None	597	55
SCMR	110	10
EACVI	58	5
SCMR + EACVI	51	5
Other	276	25

This table shows various aspects of respondents’ training, level of experience, and current professional practice/employment. The CMR sample size of each category is different since not all responders answered all questions. *CMR* cardiovascular magnetic resonance, *SCMR* Society for Cardiovascular Magnetic Resonance, *EACVI* European Association of Cardiovascular Imaging

### CMR practice type and location

Most responders (695/1014, 69%) practiced in a university/academic hospital setting, while 249/1014 (25%) reported practicing in a community/non-academic hospital (see Table 3). CMR programs were more often located in radiology departments (608/985, 62%) vs. cardiology departments (211/985, 21%), with the majority (522/606, 86%) of the programs hospital based. Only 59/333 (18%) of responders reported having an MR scanner dedicated to CMR studies, with the majority (189/333, 57%) reporting that their MR scanner was used ≤ 25% of the time for CMR studies. Clinical CMR studies performed per year were relatively evenly distributed throughout the survey question range, with 293/917 (32%) of responders reporting conducting > 1000 CMR studies per year, while 473/917 (52%) of responders reported performing ≤ 500 CMR studies per year, ranging from < 100 CMR studies per year (119/917, 13%) to > 3000 CMR studies per year (53/917, 6%). A comparison between radiology run and cardiology run practices revealed no significant difference between cardiology sites reserved > 60 min for CMR studies (26/213,12%) vs. radiology sites (132/879, 15%; p = 0.74). Rapid scanning (< 30 min time slots) was similarly low for both cardiology (9/213, 4%) and radiology (37/842, 4%; p = 0.99). In developed countries, fewer cardiology sites reserved > 60 min for CMR studies (20/151,13%) vs. radiology sites (99/481, 21%; p = 0.04). Rapid scanning (< 30 min time slots) was similarly low for both cardiology (8/151, 5%) and radiology (21/481, 4%; p = 0.65). In developing countries, a similar number of cardiology sites reserved > 60 min for CMR studies (6/28, 21%) vs. radiology sites (32/179, 18%; p = 0.65). Rapid scanning (< 30 min time slots) were similarly low for both cardiology (1/28, 4%) and radiology (16/179, 9%; p = 0.34).

**Table 3:** CMR practice type, volumes, and location

CMR practice		
Type of institution	n = 1014	%
University/academic hospital	695	69
Community/non-academic hospital	249	25
Government institution/public assistance	42	4
Research institution	28	3

The department responsible for the scanner	n = 985	%
Radiology	608	62
Cardiology	211	21
Shared	166	17

Location of CMR Program	n = 606	%
Hospital	522	86
Imaging center/outpatient facility	61	10
Both	23	4

Percentage of time of MR dedicated to CMR	n = 333	%
0–25%	189	57
26–50%	57	17
51–75%	15	5
> 75%	13	4
Dedicated scanner	59	18

Service area size (inhabitants)	n = 546	%
> 1 million	232	42
> 500,000–1 million	121	22
> 250,000–500,000	103	19
100,000–250,000	68	12
< 100,000	22	4

Clinical CMR studies performed per year	n = 917	%
< 100	119	13
101–300	193	21
301–500	161	18
501–1000	151	16
1001–2000	183	20
2001–3000	57	6
> 3000	53	6

This table shows various aspects of respondents’ CMR practice type, volumes, and location. The CMR sample size of each category is different since not all responders answered all questions. *CMR* cardiovascular magnetic resonance

### CMR main indications, referral source, and barriers

The top three referral indications were evaluation of cardiomyopathy (213/585, 36%), viability assessment (134/585, 23%), and evaluation of ischemic heart disease (97/585, 17%). Adult general cardiologists were the primary referring providers, according to 680/818 (83%) of the responders. The top 3 barriers to CMR growth were access to scanners (145/556, 26%), high cost (132/556, 24%), and competing technologies (110/556, 20%) (see Additional file 1).

### Comparison between high-volume and low-volume centers

**Fig. 2:**
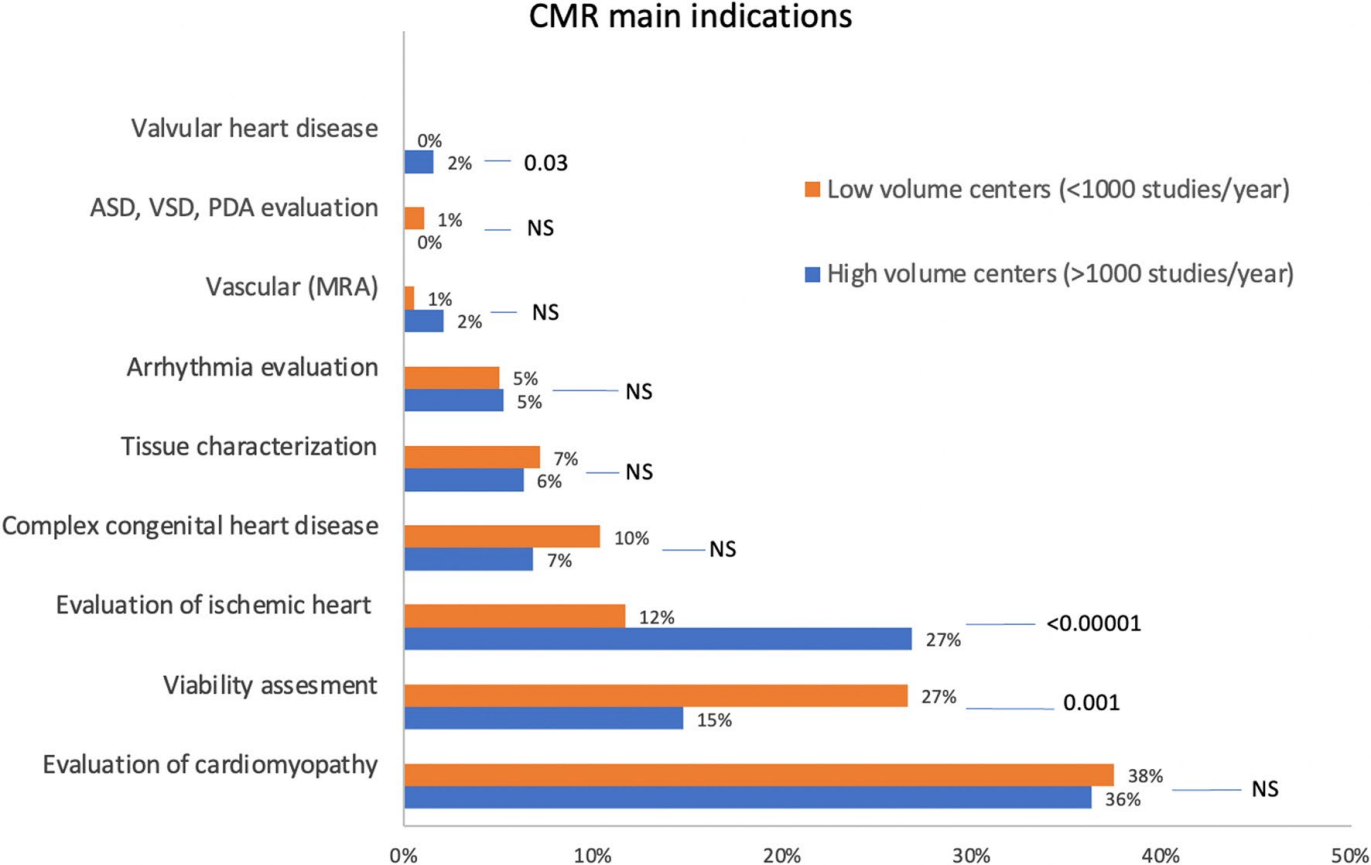
CMR main indications segregated by high vs. low volume centers (> 1000 or < / = 1000 CMR studies per year. This figure shows CMR's main indications when the respondents’ centers are divided into two groups: low-volume centers, those with a volume of < / = 1000 CMR studies per year of any kind, and large-volume centers with > 1000 CMR studies per year of any kind. *CMR* cardiovascular magnetic resonance, *NS* non-significant, *ASD* atrial septal defect, *VSD* ventricular septal defect, *PDA* patent ductus arterioles, *MRA* magnetic resonance angiography

**Fig. 3:**
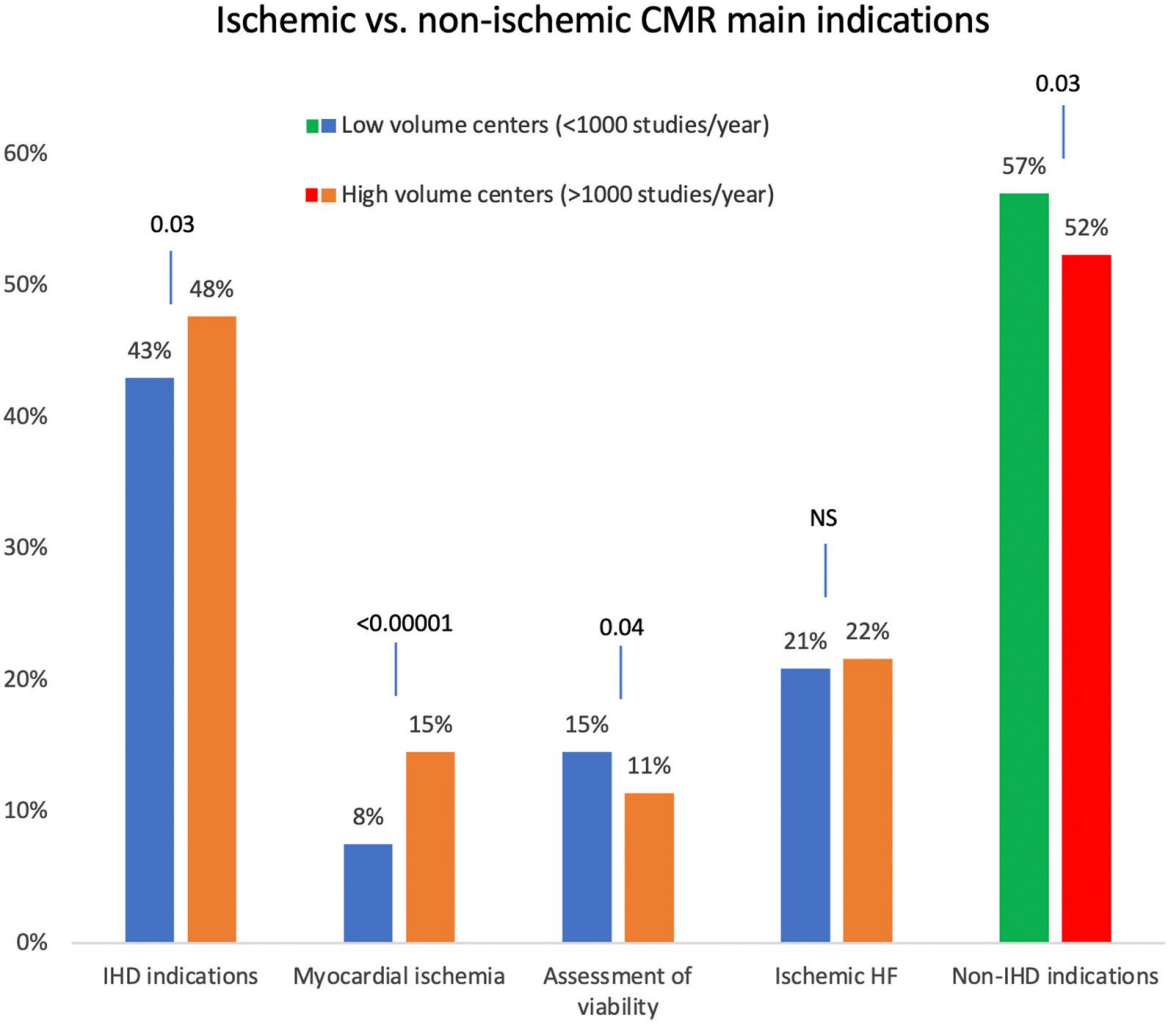
Ischemic and non-ischemic CMR main indications segregated by high vs. low volume centers (> or < / = 1000 CMR studies per year). This figure shows the distribution between ischemic and non-ischemic CMR studies and their main indications when the respondents’ centers are divided into two groups: low volume centers, those with a volume of < / = 1000 CMR studies per year of any kind, and large-volume centers with > 1000 CMR studies per year. The columns on the left labeled “IHD indications” include the following exam categories: myocardial ischemia evaluation with stress perfusion studies, the assessment of myocardial viability alone, and the evaluation of ischemic heart failure, which are also shown independently in the adjacent columns. The columns on the right (in green and red) compare all other non-ischemic heart disease-related indications. *CMR* cardiovascular magnetic resonance, *IHD* ischemic heart disease, *HF* heart failure

High-volume centers (> 1000 studies per year) were compared to low-volume centers (< / = 1000 studies per year) across CMR indications (Fig. 2). Evaluation of cardiomyopathy was the top indication in both high-volume and low-volume centers (289/954 vs 327/954; p = 0.06). The two indications that differed were the evaluation of ischemic heart disease (IHD) and viability assessment. High-volume centers were more likely to list evaluation of ischemic heart disease (e.g., stress CMR) as a primary indicator (25/183, 14%) compared to low-volume centers (35/392, 9%). This was not statistically significant overall (p = 0.08). However, there were significant differences between developed vs developing countries, where in developed countries the high-volume centers performed significantly more stress CMR (20/144, 14%) when compared to low-volume centers (17/250, 7%; p = 0.02). This was not the case in developing countries (p = 0.98). where viability assessment was more commonly listed as a primary referral reason in low-volume centers (high-volume centers: 87/293 [30%] vs. low-volume centers: 346/799 [43%]; p < 0.001). Evaluation of myocardial ischemia (including the use of stress perfusion studies) was more common in high vs. low volume centers (111/293 [38%] vs 118/799 [15%]; p < 0.001), respectively. The assessment of myocardial viability alone was similar (87/293 [30%] vs 228/799 [29%]; p = 0.70) in high vs. low volume centers. Both types of centers performed studies for evaluating ischemic heart failure at similar rates (p = 0.88). The non-IHD indications were 52% vs. 57% (p = 0.03) for high vs. low-volume centers (Fig. 3). Referring physician type was similar between high-volume and low-volume centers (data not shown).

Regarding scan slot duration reserved for CMR studies, most centers reserve 46–60 min for a CMR study (302/799, 38%) of low-volume centers and 164/293 (56%) of high-volume centers (p < 0.001), followed by 31–45-min time slots (109/799, 14%) of low-volume centers and 75/293 (26%) of high-volume centers (p < 0.001). Least frequently were the < 30-min time slots (34/799, 4%) of low-volume centers and 12/293 (4%) of high-volume centers, with no statistically significant difference between high-volume and low-volume centers (p = 0.90). In the case of long (> 60 min) scan slots, only 31/293 (11%) of high-volume centers reported using these, as compared to 127/799 (16%) of low-volume centers (p = 0.03).

**Fig. 4:**
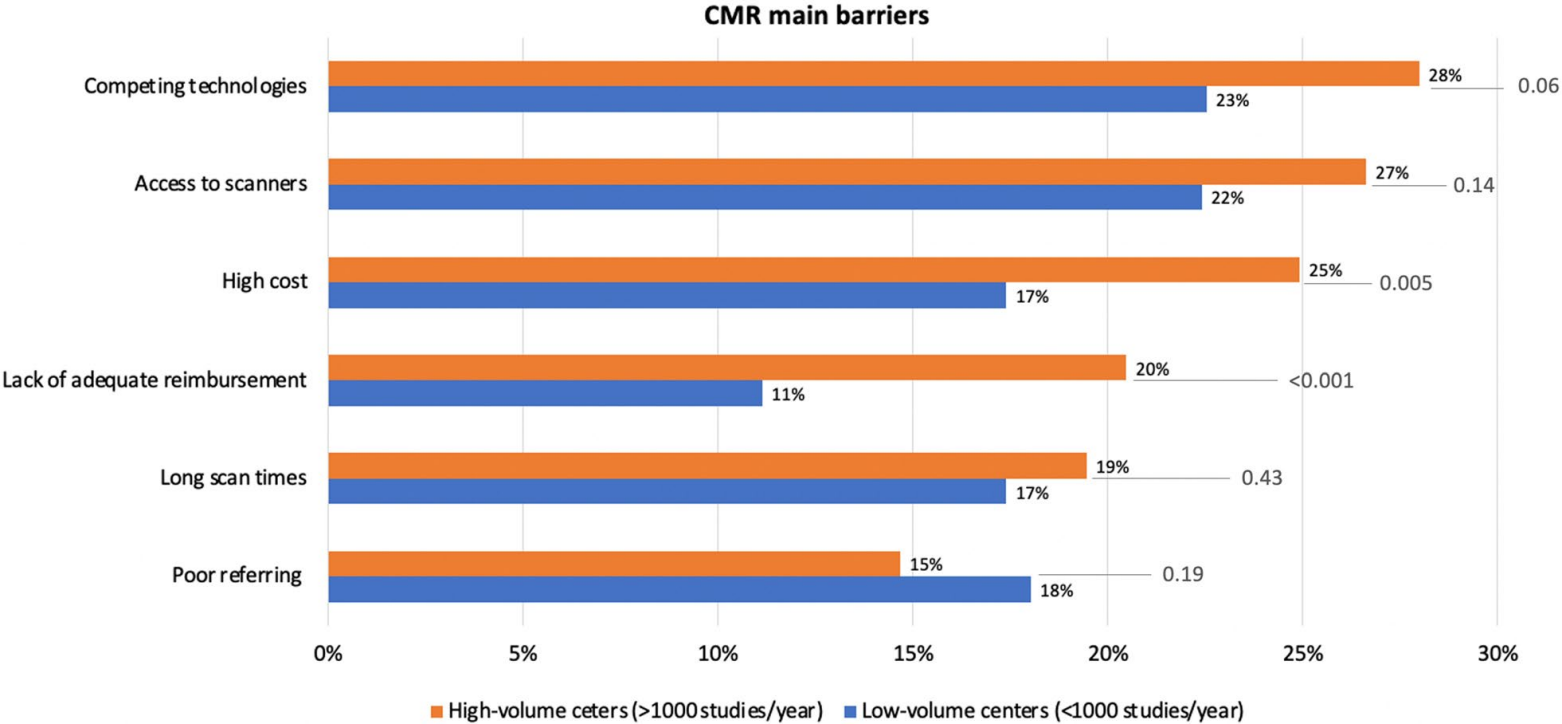
High vs. low volume centers analysis (< / = 1000 vs. > 1000 CMR studies per year)—main barriers to CMR implementation. This figure shows the CMR main barriers when the respondent centers are divided into two large groups: low-volume centers, those with a volume of < / = 1000 CMR studies per year of any kind, and those considered large-volume centers with a volume of > 1000 CMR studies per year of any kind. *CMR* cardiovascular magnetic resonance

High-volume centers were more likely to have large population service areas (122/293, 56%) reported serving areas of > 1 million people. Interestingly, 34/293 (12%) of high-volume center responders reported practicing in population centers of ≤ 500,000 inhabitants (see Additional file 1). When evaluating whether there was a difference in barriers to CMR growth between high- and low-volume centers, high-volume centers reported similar relevance to other competing technologies (82/293, [28%] vs. 180/799 [23%]; p = 0.06) and access to scanners (78/293 [27%] vs 179/799, [22%]; p = 0.14) as the most frequent obstacles. Still, only the cost (73/293 [25%] vs 139/799 [17%]; p = 0.005) and the lack of reimbursement (60/293 [20%] vs. 89/710 [11%]; p < 0.001) were a statistically significant barrier to growth (Fig. 4). In contrast, low-volume centers reported that poor referring was a more frequent impediment to their development compared for the high-volume centers. However, this difference was not significant (144/799 [18%] vs 43/293 [15%]; p = 0.19).

### Comparison between countries by economic classification

Countries and regions were classified into developing, developed, and in transition, according to the United Nations 2018 classification [17] (see Table 1). Most of the responses were from developed countries (777/1092, 71%). Lack of training was considered a barrier to the progress of CMR in developing countries (34/294, 12%), compared to 66/777 (8%) in developed countries. This difference was not significant (p = 0.12). Contrary to common belief, access to MR scanners was not reported as a significant barrier in developing countries. Only 54/294 respondents (18%) said this to be a primary barrier, compared to the developed countries, where this was reported as one of the most common barriers to progress (203/777, 26%; p = 0.007). The cost was reported as a barrier similarly in both developed (148/777, 19%) and developing (64/294, 22%; p = 0.31) countries. The most common and significant barrier reported was the competing technologies for both developed and developing countries (172/777 [22%] vs 90/294 [31%]; p = 0.003).

## Discussion

CMR is increasingly important in managing a wide range of cardiovascular conditions. This survey identified diverse places where CMR is practiced worldwide (70 countries and regions represented), with similarities and differences among practice volumes, indications, training, education, and challenges. This study presents a much broader sampling of CMR practice compared to previously published surveys and registries, such as the SCMR Global CMR Registry [20], two surveys from the UK [21, 22], and one from Canada [23]. As international efforts expand to incorporate CMR more broadly into clinical practice, these results will help inform strategies for increasing the adoption and utilization of CMR.

Some countries, such as the United Kingdom, have been very successful in increasing CMR utilization [21]. Earlier and more extensive adoption of CMR into clinical practice guidelines for chest pain evaluation and acute myocardial infarction in the UK [10] compared with the USA [9] may have impacted regional variation of CMR utilization [24, 25]. In developed and in developing countries, the three main barriers to CMR growth (in order of frequency reported) were the same: competing technologies, access to scanners, and cost.

A strength of our survey, in comparison to ones from the UK [21, 22] and Canada [23], is that we opened it to both SCMR and non-SCMR members around the world. This allowed a broader sampling of the real-world practice of CMR, with a far larger sample size and representation of countries and regions. Another strength is that our writing group comprised CMR experts worldwide, representing diverse geographic and economic regions. [19, 26] This diversity of experience allowed expert insights into the analysis and interpretation of our results.

Most responders to our survey worked in university/academic hospital systems. The majority were either adult cardiologists or radiologists. This is encouraging, as with the increasing utilization of CMR [27], there will be a growing need for reporting experts from both specialties [28]. Almost half of our responders had no formal certification in CMR; this was common in both developed and developing countries. Limited venues for formal CMR certification may reflect limited opportunities for formalized training. The development of the Certification Board of Cardiovascular Magnetic Resonance (CBCMR) (now available to practitioners worldwide) encourages the formalization of CMR training through a standardized certification [29]. However, the level of untapped interest is still to be determined, as at least in some developing countries, such as India and Mexico, practitioners are often members of their national societies but not of international organizations, such as SCMR. Inclusion and diversity in leadership positions within national and international organizations that promote the use of CMR will facilitate its growth and expansion on a global level.

This survey shows differences in CMR practice and barriers to growth both between developing and developing countries and by practice volume. A typical CMR program in this survey was an academic, radiology-based CMR program, where the reader spends, on average ≤ 25% of their time on CMR-related activities and whose primary referral source is adult cardiology. Although this appears to represent the majority, the survey included several CMR programs outside of traditional high-volume centers, as shown by the fact that 25% of responders were from centers in non-academic/community-based locations, and the majority (648/1092, 59%) of the total surveyed centers performed ≤ 500 studies/year. These findings provide a positive outlook for CMR growth beyond traditional, large academic centers and highlight the importance of training, education, and product development to support smaller but emerging programs. A more dedicated effort is also required to provide training and certification opportunities in developing countries. In addition, steps need to be taken universally to reduce the cost of and time for CMR examinations, such as with fast scanning protocols and/or contrast-free techniques. The findings of this survey will hopefully provide insights to inform planning for the Society to increase and improve CMR access in the world.

### Limitations

The survey was conducted in written English and sent to international responders; questions could have been interpreted in different ways due to language and cultural differences, which could introduce bias that may affect the accuracy of the results. Some institutions also had more than one responder which contributed to some oversampling. Blank answers in some questions resulted in variable sample sizes for some analyses. An additional limitation is the age of the study. These surveys were taken 5 years ago (2017) and before the COVID-19 pandemic, which may limit their generalizability to current practice and interim changes in practice guidelines. However, these data provide an important baseline for comparison over time.

## Conclusions

In the most extensive global assessment of the practice of CMR to date, we identified heterogeneity in training, practice models, and CMR utilization worldwide by program size and between developed and developing countries. The information obtained can help develop strategies to overcome current barriers to CMR growth and to help promote the accessibility and adoption of CMR into routine clinical practice around the world.

## Supplementary Information

**Additional file 1: Figure S1**. World maps. Color coding only indicates different regions. **Table S1.** SCMR membership distribution. **Table S2.** Respondents’ background. **Table S3**. CMR logistics. **Table S4.** CMR indications and referrals. **Table S5.** CMR main barriers. **Figure S2.** CMR main referring physicians segregated by High vs. Low volume center analysis. **Figure S3.** Community size of CMR service area segregated by High vs. Low volume center analysis.

## Data Availability

The data material is not publicly available but is available from the corresponding author upon reasonable request.

## Abbreviations

CMR: Cardiovascular magnetic resonance
SCMR: Society for Cardiovascular Magnetic Resonance
UK: United Kingdom
MRI: Magnetic resonance imaging
EACVI: European Association of Cardiovascular Imaging
USA: United States of America
WHO: World Health Organization
UN: United Nations
MRA: Magnetic resonance angiography
ASD.: Atrial septal defect
VSD: Ventricular septal defect
PDA: Patent ductus arteriosus
NS: Non-significant

## References

[1] Heidenreich PA, Bozkurt B, Aguilar D, Allen LA, Byun JJ, Colvin MM (2022). AHA/ACC/HFSA guideline for the management of heart failure: A report of the American College of Cardiology/American Heart Association joint committee on clinical practice guidelines. Circulation.

[2] Vahanian A, Beyersdorf F, Praz F, Milojevic M, Baldus S, Bauersachs J (2021). 2021 ESC/EACTS Guidelines for the management of valvular heart disease: Developed by the Task Force for the management of valvular heart disease of the European Society of Cardiology (ESC) and the European Association for Cardio-Thoracic Surgery (EACTS). Eur Heart J.

[3] Visseren FLJ, Mach F, Smulders YM, Carballo D, Koskinas KC, Bäck M (2021). 2021 ESC Guidelines on cardiovascular disease prevention in clinical practice: Developed by the Task Force for cardiovascular disease prevention in clinical practice with representatives of the European Society of Cardiology and 12 medical societies With. Eur Heart J.

[4] 2021 ESC Guidelines on cardiac pacing and cardiac resynchronization therapy. EP Europace, 2022;24(1):71–164. 10.1093/europace/euab232 PMC13179788 34455427

[5] Glikson M, Nielsen JC, Kronborg MB, Michowitz Y, Auricchio A, Barbash IM (2021). 2021 ESC Guidelines on cardiac pacing and cardiac resynchronization therapy: developed by the Task Force on cardiac pacing and cardiac resynchronization therapy of the European Society of Cardiology (ESC) With the special contribution of the European Heart Rhythm Association (EHRA). Eur Heart J.

[6] McDonagh TA, Metra M, Adamo M, Gardner RS, Baumbach A, Böhm M (2021). 2021 ESC Guidelines for the diagnosis and treatment of acute and chronic heart failure: developed by the Task Force for the diagnosis and treatment of acute and chronic heart failure of the European Society of Cardiology (ESC) With the special contribution of the Heart Failure Association (HFA) of the ESC. Eur Heart J.

[7] Al-Khatib SM, Stevenson WG, Ackerman MJ, Bryant WJ, Callans DJ, Curtis AB (2018). 2017 AHA/ACC/HRS Guideline for management of patients with ventricular arrhythmias and the prevention of sudden cardiac death: a report of the American College of Cardiology/American Heart Association task force on clinical practice guidelines and the He. J Am Coll Cardiol.

[8] Amsterdam EA, Wenger NK, Brindis RG, Casey DEJ, Ganiats TG, Holmes DRJ (2014). 2014 AHA/ACC Guideline for the management of patients with non-ST-elevation acute coronary syndromes: a report of the American College of Cardiology/American Heart Association task force on practice guidelines. J Am Coll Cardiol.

[9] Gulati M, Levy PD, Mukherjee D, Amsterdam E, Bhatt DL, Birtcher KK, et al. 2021 AHA/ACC/ASE/CHEST/SAEM/SCCT/SCMR guideline for the evaluation and diagnosis of chest pain: a report of the American College of Cardiology/American Heart Association joint committee on clinical practice guidelines. J Am Coll Cardiol. 2021;78(22): e187–e285. 10.1016/j.jacc.2021.07.053 34756653

[10] Ibanez B, James S, Agewall S, Antunes MJ, Bucciarelli-Ducci C, Bueno H (2018). 2017 ESC Guidelines for the management of acute myocardial infarction in patients presenting with ST-segment elevation: The task force for the management of acute myocardial infarction in patients presenting with ST-segment elevation of the European Soci. Eur Heart J.

[11] Knuuti J, Wijns W, Saraste A, Capodanno D, Barbato E, Funck-Brentano C (2020). 2019 ESC Guidelines for the diagnosis and management of chronic coronary syndromes. Eur Heart J.

[12] Kusumoto FM, Schoenfeld MH, Barrett C, Edgerton JR, Ellenbogen KA, Gold MR (2019). 2018 ACC/AHA/HRS guideline on the evaluation and management of patients with bradycardia and cardiac conduction delay: a report of the American College of Cardiology/American Heart Association task force on clinical practice guidelines and the Heart Rhyt. J Am Coll Cardiol.

[13] Ommen SR, Mital S, Burke MA, Day SM, Deswal A, Elliott P (2020). 2020 AHA/ACC guideline for the diagnosis and treatment of patients with hypertrophic cardiomyopathy: a report of the American College of Cardiology/American Heart Association joint committee on clinical practice guidelines. J Am Coll Cardiol. United States.

[14] Otto CM, Nishimura RA, Bonow RO, Carabello BA, Erwin JP, Gentile F (2021). ACC/AHA guideline for the management of patients with valvular heart disease: a report of the American College of Cardiology/American Heart Association joint committee on clinical practice guidelines. J Am Coll Cardiol. United States.

[15] Regitz-Zagrosek V, Roos-Hesselink JW, Bauersachs J, Blomström-Lundqvist C, Cífková R, De Bonis M (2018). 2018 ESC guidelines for the management of cardiovascular diseases during pregnancy. Eur Heart J.

[16] Shen W-K, Sheldon RS, Benditt DG, Cohen MI, Forman DE, Goldberger ZD (2017). 2017 ACC/AHA/HRS guideline for the evaluation and management of patients with syncope: a report of the American College of Cardiology/American Heart Association task force on clinical practice guidelines and the Heart Rhythm Society. J Am Coll Cardiol.

[17] Stout KK, Daniels CJ, Aboulhosn JA, Bozkurt B, Broberg CS, Colman JM (2019). 2018 AHA/ACC guideline for the management of adults with congenital heart disease: a report of the American College of Cardiology/American Heart Association task force on clinical practice guidelines. J Am Coll Cardiol.

[18] Mehta LS, Warnes CA, Bradley E, Burton T, Economy K, Mehran R (2020). Cardiovascular considerations in caring for pregnant patients: a scientific statement from the American Heart Association. Circulation.

[19] Zhenmin L, Kituyi M, Songwe V, Algayerova O, Bárcena A, Akhthar S, et al. World economic situation prospects 2018. First. New York: United Nations Publication; 2018. eISBN: 978-92-1-362882-9.

[20] Kwong RY, Petersen SE, Schulz-Menger J, Arai AE, Bingham SE, Chen Y (2017). The global cardiovascular magnetic resonance registry (GCMR) of the Society for Cardiovascular Magnetic Resonance (SCMR): its goals, rationale, data infrastructure, and current developments. J Cardiovasc Magn Reson.

[21] Antony R, Daghem M, McCann GP, Daghem S, Moon J, Pennell DJ (2011). Cardiovascular magnetic resonance activity in the United Kingdom: a survey on behalf of the British Society of Cardiovascular Magnetic Resonance. J Cardiovasc Magn Reson.

[22] Keenan NG, Captur G, Mccann GP, Berry C, Myerson SG, Fairbairn T (2021). Regional variation in cardiovascular magnetic resonance service delivery across the UK. Heart.

[23] Roifman I, Paterson DI, Jimenez-Juan L, Friedrich MG, Howarth AG, Wintersperger BJ (2018). The state of cardiovascular magnetic resonance imaging in Canada: results from the CanSCMR Pan-Canadian survey. Can J Cardiol.

[24] Ferrari VA, Whitman B, Blankenship JC, Budoff MJ, Costa M, Weissman NJ (2014). Cardiovascular imaging payment and reimbursement systems: understanding the past and present in order to guide the future. JACC Cardiovasc Imaging.

[25] Petersen SE, Friebel R, Ferrari V, Han Y, Aung N, Kenawy A (2021). Recent trends and potential drivers of non-invasive cardiovascular imaging use in the United States of America and England. Front Cardiovasc Med.

[26] Department for General Assembly and Conference Management. United Nations regional groups of member states. https://www.un.org/depts/DGACM/RegionalGroups.shtml.

[27] Goldfarb JW, Weber J. Trends in cardiovascular MRI and CT in the U.S. Medicare Population from 2012 to 2017. 2021. 10.1148/ryct.2021200112 PMC7977977 33778651

[28] Bierhals AJ (2021). Data that support removing the mysticism for radiologist’s performance of cardiac CT and MRI. Radiol Cardiothorac Imaging.

[29] APCA Alliance for Physician Certification & Advancement. http://www.apca.org/certifications-examinations/CBNC-And-CBCCT/Pages/Certification-Board-of-Cardiovascular-Magnetic-Resonance.aspx.

