# Supplementary material for: Worldwide variation in cardiovascular magnetic resonance practice models
Source: J Cardiovasc Magn Reson. 2023 Jul 3;25:38. doi: 10.1186/s12968-023-00948-7 (PMC10316597; doi:10.1186/s12968-023-00948-7)
Supplement: Supplementary file 1 — Additional file 1: Figure S1. World maps. Color coding only indicates different regions. Table S1. SCMR membership distribution. Table S2. Respondents’ background. Table S3. CMR logistics. Table S4. CMR indications and referrals. Table S5. CMR main barriers. Figure S2. CMR main referring physicians segregated by High vs. Low volume center analysis. Figure S3. Community size of CMR service area segregated by High vs. Low volume center analysis. [file 12968_2023_948_MOESM1_ESM.docx]

**SUPPLEMENT MATERIAL**

**METHODS:**

**FULL LIST OF CONCEPTS SURVEYED:**

Demographics:

- Gender
- Age range
- Country of origin
- Primary background
- Current position
- Practice type
- SCMR membership status
- Training analysis
  - Formal CMR training
  - Level of expertise
  - Years of experience
  - Official certifications
- CMR logistics
  - Size of the CMR program service area
  - Type of Institution
  - Department responsible for CMR scanner
  - Type of facility for primary location of CMR program
  - Years providing CMR service
  - Number of clinical CMR studies
  - Percentage of total scanner operational time designated for CMR.
  - Time reserved for CMR.
  - Percentage of time supervising and/or reading CMR studies
- Specific components of a routine CMR study
  - MRA as part of CMR program
  - Stress CMR.
- Use of MRI vendor-proprietary or third-party post-processing software
- Growth and barriers
  - Main barriers to CMR at the institutional level
  - Top referring physicians
  - Top referral indications
  - Competing imaging technologies
  - Current and future technical development
  - Most beneficial improvements in CMR.
  - Potential routine clinical use of 4D flow and strain
  - The clinical application that would be a game-changer
  - Next promising applications
- Costs and competing technologies
  - Costs of CMR compared against Echocardiogram and other modalities, including SPECT, PET, CT perfusion, CCTA, and conventional angiography.
  - Limitation of CMR costs compared with other imaging modalities for its use

**Survey questions.**

**Survey one.**

1. **Gender:**
   1. Male
   2. Female
2. **Age Range:**
   1. 21-30
   2. 31-40
   3. 41-50
   4. 51-60
   5. 61-70
   6. >70
3. **Type of Institution:**
   1. University/Academic Hospital
   2. Community/Non-academic
   3. Public Assistance/Charity
   4. Research Institution
   5. Government Institution
4. **Background:**
   1. Attending/Staff Physician
   2. Technologist
   3. Physicist
   4. Nurse
   5. Physician Trainee/Fellow
   6. Student
5. **Primary Specialty (Physicians Only):**
   1. Radiology
   2. Cardiology
   3. Nuclear Medicine
6. **Primary Income for Your Practice (Physicians Only):**
   1. Salary Positions
   2. Private/Insurance
   3. Government
   4. Research
7. **Level of Your Expertise (Physicians Only):**
   1. Medical Director of CMR (Department, Service, Area, Lab, etc)
   2. Participating Faculty/Staff of CMR Facility
   3. CMR Fellow
8. **Region of the World:**
   1. North America (US Canada Mexico)
   2. Central America
   3. South America
   4. Western Europe
   5. Eastern Europe
   6. Middle East
   7. Eastern Asia
   8. Southern Asia
   9. Japan
   10. Asia Pacific
   11. Northern Africa
   12. Central Africa
   13. Southeastern Africa
   14. Australia/New Zealand
9. **Country:**
   1. Afghanistan
   2. Akrotiri
   3. Albania
   4. Algeria
   5. American Samoa
   6. Andorra
   7. Angola
   8. Anguilla
   9. Antarctica
   10. Antigua and Barbuda
   11. Argentina
   12. Armenia
   13. Aruba
   14. Ashmore and Cartier Islands
   15. Australia
   16. Austria
   17. Azerbaijan
   18. The Bahamas
   19. Bahrain
   20. Bangladesh
   21. Barbados
   22. Bassas da India
   23. Belarus
   24. Belgium
   25. Belize
   26. Benin
   27. Bermuda
   28. Bhutan
   29. Bolivia
   30. Bosnia and Herzegovina
   31. Botswana
   32. Bouvet Island
   33. Brazil
   34. British Indian Ocean Territory
   35. British Virgin Islands
   36. Brunei
   37. Bulgaria
   38. Burkina Faso
   39. Burma
   40. Burundi
   41. Cambodia
   42. Cameroon
   43. Canada
   44. Cape Verde
   45. Cayman Islands
   46. Central African Republic
   47. Chad
   48. Chile
   49. China
   50. Christmas Island
   51. Clipperton Island
   52. Cocos (Keeling) Islands
   53. Colombia
   54. Comoros
   55. Congo, Democratic Republic of the
   56. Congo, Republic of the
   57. Cook Islands
   58. Coral Sea Islands
   59. Costa Rica
   60. Cote d'Ivoire
   61. Croatia
   62. Cuba
   63. Cyprus
   64. Czech Republic
   65. Denmark
   66. Dhekelia
   67. Djibouti
   68. Dominica
   69. Dominican Republic
   70. Ecuador
   71. Egypt
   72. El Salvador
   73. Equatorial Guinea
   74. Eritrea
   75. Estonia
   76. Ethiopia
   77. Europa Island
   78. Falkland Islands (Islas Malvinas)
   79. Faroe Islands
   80. Fiji
   81. Finland
   82. France
   83. French Guiana
   84. French Polynesia
   85. French Southern and Antarctic Lands
   86. Gabon
   87. Gambia, The
   88. Gaza Strip
   89. Georgia
   90. Germany
   91. Ghana
   92. Gibraltar
   93. Glorioso Islands
   94. Greece
   95. Greenland
   96. Grenada
   97. Guadeloupe
   98. Guam
   99. Guatemala
   100. Guernsey
   101. Guinea
   102. Guinea-Bissau
   103. Guyana
   104. Haiti
   105. Heard Island and McDonald Islands
   106. Holy See (Vatican City)
   107. Honduras
   108. Hong Kong
   109. Hungary
   110. Iceland
   111. India
   112. Indonesia
   113. Iran
   114. Iraq
   115. Ireland
   116. Isle of Man
   117. Israel
   118. Italy
   119. Jamaica
   120. Jan Mayen
   121. Japan
   122. Jersey
   123. Jordan
   124. Juan de Nova Island
   125. Kazakhstan
   126. Kenya
   127. Kiribati
   128. Korea, North
   129. Korea, South
   130. Kuwait
   131. Kyrgyzstan
   132. Laos
   133. Latvia
   134. Lebanon
   135. Lesotho
   136. Liberia
   137. Libya
   138. Liechtenstein
   139. Lithuania
   140. Luxembourg
   141. Macau
   142. Macedonia
   143. Madagascar
   144. Malawi
   145. Malaysia
   146. Maldives
   147. Mali
   148. Malta
   149. Marshall Islands
   150. Martinique
   151. Mauritania
   152. Mauritius
   153. Mayotte
   154. Mexico
   155. Micronesia, Federated States of
   156. Moldova
   157. Monaco
   158. Mongolia
   159. Montserrat
   160. Morocco
   161. Mozambique
   162. Namibia
   163. Nauru
   164. Navassa Island
   165. Nepal
   166. Netherlands
   167. Netherlands Antilles
   168. New Caledonia
   169. New Zealand
   170. Nicaragua
   171. Niger
   172. Nigeria
   173. Niue
   174. Norfolk Island
   175. Northern Mariana Islands
   176. Norway
   177. Oman
   178. Pakistan
   179. Palau
   180. Panama
   181. Papua New Guinea
   182. Paracel Islands
   183. Paraguay
   184. Peru
   185. Philippines
   186. Pitcairn Islands
   187. Poland
   188. Portugal
   189. Puerto Rico
   190. Qatar
   191. Reunion
   192. Romania
   193. Russia
   194. Rwanda
   195. Saint Helena
   196. Saint Kitts and Nevis
   197. Saint Lucia
   198. Saint Pierre and Miquelon
   199. Saint Vincent and the Grenadines
   200. Samoa
   201. San Marino
   202. Sao Tome and Principe
   203. Saudi Arabia
   204. Senegal
   205. Serbia and Montenegro
   206. Seychelles
   207. Sierra Leone
   208. Singapore
   209. Slovakia
   210. Slovenia
   211. Solomon Islands
   212. Somalia
   213. South Africa
   214. South Georgia and the South Sandwich Islands
   215. Spain
   216. Spratly Islands
   217. Sri Lanka
   218. Sudan
   219. Suriname
   220. Svalbard
   221. Swaziland
   222. Sweden
   223. Switzerland
   224. Syria
   225. Taiwan
   226. Tajikistan
   227. Tanzania
   228. Thailand
   229. Timor-Leste
   230. Togo
   231. Tokelau
   232. Tonga
   233. Trinidad and Tobago
   234. Tromelin Island
   235. Tunisia
   236. Turkey
   237. Turkmenistan
   238. Turks and Caicos Islands
   239. Tuvalu
   240. Uganda
   241. Ukraine
   242. United Arab Emirates
   243. United Kingdom
   244. United States
   245. Uruguay
   246. Uzbekistan
   247. Vanuatu
   248. Venezuela
   249. Vietnam
   250. Virgin Islands
   251. Wake Island
   252. Wallis and Futuna
   253. West Bank
   254. Western Sahara
   255. Yemen
   256. Zambia
   257. Zimbabwe
10. **Type of City of Your Institution:**
    1. Capital City
    2. Large City in Your Country
    3. Small/Medium City in Your Country
    4. Rural City in Your Country
11. **Medical Facility Type:**
    1. Research
    2. Advanced Medical Attention
    3. Basic Medical Attention
12. **CMR Study Payment. Check all that apply:**
    1. Patient's Out of Pocket
    2. Third Party Payer (Insurance/Health Maintenance Organization)
    3. Social Security
    4. Government Funded
    5. Foundation Funded
    6. Research
13. **What is Your Annual Income?**
    1. Less than $100,000 USD
    2. $100,001 - $300,000 USD
    3. $300,001 - $500,000 USD
    4. More than $500,000 USD
14. **What is the Percentage of the Total Operational Time Designated for CMR?**
    1. 0-25%
    2. 26-50%
    3. 51-75%
    4. >75%
    5. Dedicated Scanner to CMR

1. **Department Responsible for the CMR Scanner:**
   1. Radiology and Imaging
   2. Cardiology
   3. Shared Between Radiology and Cardiology
2. **Is there a Primary Economic Barrier to Implementation of CMR? Check all that apply:**
   1. High Out of Pocket Cost to Patient
   2. Lack of Reimbursement
   3. Access to Care Because of Inadequate Funding/Rescources
   4. Costs of CMR Study Compared With Other Current Imaging Modalities
   5. Not Covered by Third-party Payment (Insurance/HMO)
   6. Long Waiting Lists for Social Security Services
   7. Insufficient Government Funding for Clinical or Research Purposes
3. **Is Language a Major Barrier to Correctly Understand and Implement the CMR Literature?**
   1. Yes
   2. No
   3. Maybe, but I had not considered it as a potential barrier to CMR growth in my Country before.
4. **Would having key papers, guidelines, appropriate criteria, statement position documents, and key research papers translated into your local Language improve your ability to deliver CMR to your community?**
   1. Yes
   2. No
   3. Maybe, but I had not considered it as a potential barrier to CMR growth in my Country before.
5. **Is complexity of terminology a limitation for broader growth of CMR?**
   1. Yes
   2. No
   3. Maybe, but I had not considered it as a potential barrier to CMR growth in my Country before.
6. **Do you think it would be useful and a meaningful effort to have key papers, guidelines, appropriate criteria, statement position documents and key research papers written in simpler terms?**
   1. Yes
   2. No
   3. Maybe, but I had not considered it as a potential barrier to CMR growth in my Country before.
7. **Did you know that a proposal to simplify CMR terminology exists?**
   1. Yes
   2. No
   3. I don't know
8. **Do you find the proposal of simpler, globally-consistent CMR terminology useful?**
   1. Yes
   2. No
   3. I don't know
9. **Do you think all commercial brands should unify the acquisition parameters in just one unique technical term instead of having many different names for the same one?**
   1. Yes
   2. No
   3. I don't know
10. **The cost of non-contrast functional CMR compared to Transthoracic Echo is?**
    1. Less Expensive
    2. More Expensive
    3. Similar
    4. I don't know
11. **The cost of non-contrast functional CMR compared to Transesophageal Echo is?**
    1. Less Expensive
    2. More Expensive
    3. Similar
    4. I don't know
12. **The cost of contrast stress perfusion CMR compared to Dobutamine Echo is?**
    1. Less Expensive
    2. More Expensive
    3. Similar
    4. I don't know
13. **The cost of contrast stress perfusion CMR compared to Nuclear (SPECT) is?**
    1. Less Expensive
    2. More Expensive
    3. Similar
    4. I don't know
14. **The cost of contrast stress perfusion CMR compared to Nuclear (PET) is?**
    1. Less Expensive
    2. More Expensive
    3. Similar
    4. I don't know
15. **The cost of contrast CMR (viability) compared to Dobutamine Echo is?**
    1. Less Expensive
    2. More Expensive
    3. Similar
    4. I don't know
16. **The cost of contrast CMR (viability) compared to Nuclear (SPECT) is?**
    1. Less Expensive
    2. More Expensive
    3. Similar
    4. I don't know
17. **The cost of contrast CMR (viability)compared to Nuclear (PET) is?**
    1. Less Expensive
    2. More Expensive
    3. Similar
    4. I don't know
18. **The cost of contrast stress perfusion CMR compared to CCTA is?**
    1. Less Expensive
    2. More Expensive
    3. Similar
    4. I don't know

1. **Is the cost of contrast CMR for tissue characterization within the range of other similar imaging modalities?**
   1. Less Expensive
   2. More Expensive
   3. Similar
   4. I don't know

1. **Country of Training:**
   1. Afghanistan
   2. Akrotiri
   3. Albania
   4. Algeria
   5. American Samoa
   6. Andorra
   7. Angola
   8. Anguilla
   9. Antarctica
   10. Antigua and Barbuda
   11. Argentina
   12. Armenia
   13. Aruba
   14. Ashmore and Cartier Islands
   15. Australia
   16. Austria
   17. Azerbaijan
   18. The Bahamas
   19. Bahrain
   20. Bangladesh
   21. Barbados
   22. Bassas da India
   23. Belarus
   24. Belgium
   25. Belize
   26. Benin
   27. Bermuda
   28. Bhutan
   29. Bolivia
   30. Bosnia and Herzegovina
   31. Botswana
   32. Bouvet Island
   33. Brazil
   34. British Indian Ocean Territory
   35. British Virgin Islands
   36. Brunei
   37. Bulgaria
   38. Burkina Faso
   39. Burma
   40. Burundi
   41. Cambodia
   42. Cameroon
   43. Canada
   44. Cape Verde
   45. Cayman Islands
   46. Central African Republic
   47. Chad
   48. Chile
   49. China
   50. Christmas Island
   51. Clipperton Island
   52. Cocos (Keeling) Islands
   53. Colombia
   54. Comoros
   55. Congo, Democratic Republic of the
   56. Congo, Republic of the
   57. Cook Islands
   58. Coral Sea Islands
   59. Costa Rica
   60. Cote d'Ivoire
   61. Croatia
   62. Cuba
   63. Cyprus
   64. Czech Republic
   65. Denmark
   66. Dhekelia
   67. Djibouti
   68. Dominica
   69. Dominican Republic
   70. Ecuador
   71. Egypt
   72. El Salvador
   73. Equatorial Guinea
   74. Eritrea
   75. Estonia
   76. Ethiopia
   77. Europa Island
   78. Falkland Islands (Islas Malvinas)
   79. Faroe Islands
   80. Fiji
   81. Finland
   82. France
   83. French Guiana
   84. French Polynesia
   85. French Southern and Antarctic Lands
   86. Gabon
   87. Gambia, The
   88. Gaza Strip
   89. Georgia
   90. Germany
   91. Ghana
   92. Gibraltar
   93. Glorioso Islands
   94. Greece
   95. Greenland
   96. Grenada
   97. Guadeloupe
   98. Guam
   99. Guatemala
   100. Guernsey
   101. Guinea
   102. Guinea-Bissau
   103. Guyana
   104. Haiti
   105. Heard Island and McDonald Islands
   106. Holy See (Vatican City)
   107. Honduras
   108. Hong Kong
   109. Hungary
   110. Iceland
   111. India
   112. Indonesia
   113. Iran
   114. Iraq
   115. Ireland
   116. Isle of Man
   117. Israel
   118. Italy
   119. Jamaica
   120. Jan Mayen
   121. Japan
   122. Jersey
   123. Jordan
   124. Juan de Nova Island
   125. Kazakhstan
   126. Kenya
   127. Kiribati
   128. Korea, North
   129. Korea, South
   130. Kuwait
   131. Kyrgyzstan
   132. Laos
   133. Latvia
   134. Lebanon
   135. Lesotho
   136. Liberia
   137. Libya
   138. Liechtenstein
   139. Lithuania
   140. Luxembourg
   141. Macau
   142. Macedonia
   143. Madagascar
   144. Malawi
   145. Malaysia
   146. Maldives
   147. Mali
   148. Malta
   149. Marshall Islands
   150. Martinique
   151. Mauritania
   152. Mauritius
   153. Mayotte
   154. Mexico
   155. Micronesia, Federated States of
   156. Moldova
   157. Monaco
   158. Mongolia
   159. Montserrat
   160. Morocco
   161. Mozambique
   162. Namibia
   163. Nauru
   164. Navassa Island
   165. Nepal
   166. Netherlands
   167. Netherlands Antilles
   168. New Caledonia
   169. New Zealand
   170. Nicaragua
   171. Niger
   172. Nigeria
   173. Niue
   174. Norfolk Island
   175. Northern Mariana Islands
   176. Norway
   177. Oman
   178. Pakistan
   179. Palau
   180. Panama
   181. Papua New Guinea
   182. Paracel Islands
   183. Paraguay
   184. Peru
   185. Philippines
   186. Pitcairn Islands
   187. Poland
   188. Portugal
   189. Puerto Rico
   190. Qatar
   191. Reunion
   192. Romania
   193. Russia
   194. Rwanda
   195. Saint Helena
   196. Saint Kitts and Nevis
   197. Saint Lucia
   198. Saint Pierre and Miquelon
   199. Saint Vincent and the Grenadines
   200. Samoa
   201. San Marino
   202. Sao Tome and Principe
   203. Saudi Arabia
   204. Senegal
   205. Serbia and Montenegro
   206. Seychelles
   207. Sierra Leone
   208. Singapore
   209. Slovakia
   210. Slovenia
   211. Solomon Islands
   212. Somalia
   213. South Africa
   214. South Georgia and the South Sandwich Islands
   215. Spain
   216. Spratly Islands
   217. Sri Lanka
   218. Sudan
   219. Suriname
   220. Svalbard
   221. Swaziland
   222. Sweden
   223. Switzerland
   224. Syria
   225. Taiwan
   226. Tajikistan
   227. Tanzania
   228. Thailand
   229. Timor-Leste
   230. Togo
   231. Tokelau
   232. Tonga
   233. Trinidad and Tobago
   234. Tromelin Island
   235. Tunisia
   236. Turkey
   237. Turkmenistan
   238. Turks and Caicos Islands
   239. Tuvalu
   240. Uganda
   241. Ukraine
   242. United Arab Emirates
   243. United Kingdom
   244. United States
   245. Uruguay
   246. Uzbekistan
   247. Vanuatu
   248. Venezuela
   249. Vietnam
   250. Virgin Islands
   251. Wake Island
   252. Wallis and Futuna
   253. West Bank
   254. Western Sahara
   255. Yemen
   256. Zambia
   257. Zimbabwe
2. **In routine clinical practice, when there is discrepancy between Echo and CMR findings, which result do the referring physician believe as correct?**
   1. Echo
   2. CMR
   3. I don't know
3. **In routine clinical practice, when there is discrepancy between SPECT and CMR findings, which result do the referring physician believe as correct?**
   1. SPECT
   2. CMR
   3. I don't know
4. **In routine clinical practice, when there is discrepancy between PET and CMR findings, which result do the referring physician believe as correct?**
   1. PET
   2. CMR
   3. I don't know
5. **In your clinical practice, does the radiologist show interest and respect for CMR?**
   1. Yes
   2. No
   3. I don't know
6. **In your clinical practice, does the cardiologist show interest and respect for CMR?**
   1. Yes
   2. No
   3. I don't know

1. **If you are working in a non-dedicated CMR scanner, do you have difficulty scheduling patients?**
   1. Yes
   2. No
   3. I don't know
2. **What kind of CMR training did the technologist at your facility receive?**
   1. Attended technologist's track session at a SCMR meeting
   2. Other formal training
   3. No formal training; I or another physician at my institution trained the technologist
   4. No formal training
   5. I don't know

1. **Do you have residents/fellows rotating with you in CMR as part of their General Radiology and/or Cardiology training?**
   1. Yes
   2. No
   3. I don't know
2. **Do you have a formal CMR training program with fellows in your Institution?**
   1. Yes
   2. No
   3. I don't know

1. **Are you invited to participate in different types of Institutional Scientific Sessions (Grand Rounds, Lectures, Cases Reviews, Teaching, etc) in diverse areas such as Internal Medicine, Cardiology, Radiology, etc?**
2. Yes
3. No
4. I don't know
5. **Do you have IRB–approved protocols involving CMR?**
   1. Yes
   2. No
   3. I don't know
6. **Do you have intramural or extramural research support for basic and/or Clinical CMR research?**
   1. Yes
   2. No
   3. I don't know

1. **Where do you publish your research?**
   1. In a local Journal in your Language
   2. In an International Journal in English
   3. In local and International Journal in local Language and in English
   4. I do not publish CMR
2. **When you submit your research to an International Journal or Website Clinical Cases Site, do you get feedback regarding the following recommendations? Check all that apply.**
   1. Language recommendations
   2. Image quality recommendations
   3. CMR Technical recommendations
   4. Statistical analysis recommendations
   5. Reference recommendations
3. **How many CMR studies does your Institution perform yearly?**
   1. <100
   2. 101-300
   3. 301-500
   4. 501-1000
   5. >1000
4. **Which type of CMR studies does your Institution perform? Check all that apply.**
   1. Vasodilator stress perfusion
   2. Viability
   3. Ventricular function
   4. Dobutamine stress
   5. Simple Congenital Heart Disease
   6. Complex Congenial Heart Disease
   7. Vascular (MRA)
   8. Tissue characterization (myocarditis)
   9. Tissue characterization (pericardial disease)
   10. Tissue characterization (cardiac mases)
   11. Tissue characterization (cardiomyopathies)
   12. Valvular heart disease
   13. Other
5. **Is CMR part of the training of General Cardiology in your Institution?**
   1. Yes
   2. No
   3. There is no General Cardiology training program in my Institution
   4. NA
6. **Is CMR part of the training of Internal Medicine or similar specialties in your Institution?**
   1. Yes
   2. No
   3. NA
7. **How do you grade the overall quality of your CMR local Meetings?**
   1. Poor
   2. Fair
   3. Good
   4. Excellent

1. **How do you grade the overall quality of your CMR Regional Meetings?**
   1. Poor
   2. Fair
   3. Good
   4. Excellent
2. **How do you grade the overall quality of your CMR National Meetings?**
   1. Poor
   2. Fair
   3. Good
   4. Excellent

1. **Do you have a significant participation of CMR (at least 10% of the full Scientific Program) within your General Cardiology National Meetings?**
   1. Yes
   2. No
   3. I don't know

1. **Do you have a significant participation of CMR (at least 10% of the full Scientific Program) within your General Radiology National Meetings?**
   1. Yes
   2. No
   3. I don't know

1. **What type of physician specialist perform CMR at your Institution?**
   1. Cardiologist
   2. Radiologist
   3. Nuclear Cardiologist
   4. Interventional Cardiologist
   5. Other
2. **What kind of training does your CMR physician have?**
   1. Informal
   2. 1 month in a specialize Center
   3. 1-3 months in a specialize Center
   4. 3-6 months in a specialize Center
   5. 6-12 months in a specialize Center
   6. More than 12 months in a specialize Center
3. **Which of the following do you consider as a major technical limitation for CMR at your Institution? Check all that apply.**
   1. Patient cooperation
   2. Breath-hold duration
   3. EKG gating/arrhythmia
   4. Additional hardware availability (i.e. Physiological monitoring, MR compatible infusion pumps, Anesthesia carts, etc)
   5. Very limited time for CMR studies in a non-dedicated scanner
   6. Technologist limited expertise
   7. Nurse limited expertise
4. **How often you have to deal with Claustrophobia in your patients?**
   1. <25% of cases
   2. 25-50% of cases
   3. 51-75% of cases
   4. >75% of cases
5. **How do you deal with Claustrophobia at your Institution?**
   1. Supportive measures
   2. Use oral anxiolytic medication
   3. Use IV anxiolysis, sedation or anesthesia
6. **What percentage of CMR exams in Claustrophobic patients have to be cancelled?**
   1. <5%
   2. 6-10%
   3. 11-50%
   4. >50%
7. **Do you think all personnel involved in scanning the patients have enough physics knowledge to overcome artifacts and technical related problems?**
   1. Yes
   2. No
   3. I don't think physics knowledge is needed
   4. I don't know
8. **Do you consider that referring physicians at your Institution choose a specific imaging modality taking into account the use and dose of radiation?**
   1. Yes
   2. No
   3. I don't know
9. **In Pediatric population, what is most important at your Institution, the need of Anesthesia or the use of radiation to choose the imaging modality? Check all that apply.**
   1. The need of Anesthesia for CMR
   2. The use of Anesthesia for CT
   3. We prefer CT since we are able to do it without anesthesia
   4. We prefer CMR since we are able to do it without anesthesia
   5. We choose only based of the information we need and the appropriateness criteria-current guidelines
10. **Who is the referring physician? Check all that apply.**
    1. Adult/general Cardiologist who does not do imaging
    2. Pediatric Cardiologist
    3. Interventional Cardiologist
    4. Electrophysiologist
    5. Cardiologist - Echo expert
    6. Cardiologist - CT expert
    7. Cardiologist - Nuclear expert
    8. Internist/Family practitioner
    9. Emergency Medicine
    10. ICU specialist
    11. Anesthesiologist
    12. Cardiovascular surgeon
11. **What is the accessibility of CMR facility/expert physician at your Institution?**
    1. Not available
    2. Available but only weekdays
    3. Available weekdays and occasional weekend/holiday access
    4. Available 24 hours/day, 7 days/week including holidays
12. **What is the accessibility of Echo facility/expert physician at your Institution?**
    1. Not available
    2. Available but only weekdays
    3. Available weekdays and occasional weekend/holiday access
    4. Available 24 hours/day, 7 days/week including holidays
13. **What is the accessibility of Nuclear facility/expert physician at your Institution?**
    1. Not available
    2. Available but only weekdays
    3. Available weekdays and occasional weekend/holiday access
    4. Available 24 hours/day, 7 days/week including holidays
14. **What is the accessibility of CT facility/expert physician at your Institution?**
    1. Not available
    2. Available but only weekdays
    3. Available weekdays and occasional weekend/holiday access
    4. Available 24 hours/day, 7 days/week including holidays
15. **What field strength scanner(s) do you use for CMR? Check all that apply.**
    1. 1 T
    2. 1.5 T
    3. 3 T
    4. >3 T
    5. I don't know
16. **What brand(s) of CMR scanner does your Institution use? Check all that apply.**
    1. GE
    2. Siemens
    3. Philips
    4. Toshiba
    5. Other
17. **What type of scanner access does your Institution have for CMR? Check all that apply.**
    1. Clinical
    2. Research
18. **Is there an established anesthesia protocol for CMR at your Institution?**
    1. Yes
    2. No
    3. I don't know
19. **Is there a QA by peers program at your Institution?**
    1. Yes
    2. No
    3. I don't know
20. **The QA program by peers is performed within your own Institution or in collaboration with another Institution?**
    1. We don't have a QA program by peers
    2. Is performed within our Institution
    3. Is performed in collaboration with another Institution in the same Country
    4. Is performed in collaboration with another Institution in a different Country
    5. I don´t know
21. **Is your CMR program accredited by an external organization?**
    1. No
    2. Yes, IAC
    3. Yes, ACR
    4. Yes, other entity

1. **Of the physicians doing CMR at your Institution, how many maintain Level 3 designation from SCMR?**
   1. 0
   2. 1
   3. 2-3
   4. >3
2. **Of the physicians doing CMR at your Institution, how many maintain Level 2 designation from SCMR?**
   1. 0
   2. 1
   3. 2-3
   4. >3
3. **Please enter your Name and Email Address below.**
   1. Open-Ended Response

**Survey two.**

1. **Type of SCMR Membership:**
   1. Associate Member
   2. Full Member
   3. Not a member of SCMR
   4. Technologist Member
   5. Trainee Member
2. **Gender:**
   1. Female
   2. Male
3. **Age Range:**
   1. </=30 years
   2. 31-40
   3. 41-50
   4. 51-60
   5. >60 years
4. **Describe your current primary practice type/position:**
   1. Employed physician (hospital, government, academic)
   2. Private practice/Independent physician
   3. Scientist (non-clinician)
   4. Student/Trainee
   5. Technologist/Radiographer
   6. Other (please specify)
5. **Describe your current primary practice type/position, if other please specify:**
   1. Open-Ended Response
6. **Primary Focus/ Specialty:**
   1. Radiology (adult)
   2. Radiology (pediatric)
   3. Cardiology (adult)
   4. Cardiology (pediatric)
   5. Scientist (non-clinician)
   6. Technologist
   7. Medical Industry
   8. Other (please specify) Open-Ended Response
7. **What % of your time do you spend supervising/reading clinical CMR studies?**
   1. <10%
   2. 11-25%
   3. 26-50%
   4. 51-75%
   5. >75%
   6. Not applicable
8. **What is your role in your institutions CMR program (reading physicians only):**
   1. CMR Fellow
   2. Medical Director of CMR Facility (Department, Service, Area, Lab, etc.)
   3. Participating Faculty / Reading Staff of CMR facility
   4. Not applicable
   5. Other (please specify) Open-Ended Response
9. **Country:**
   1. Afghanistan
   2. Akrotiri
   3. Albania
   4. Algeria
   5. American Samoa
   6. Andorra
   7. Angola
   8. Anguilla
   9. Antarctica
   10. Antigua and Barbuda
   11. Argentina
   12. Armenia
   13. Aruba
   14. Ashmore and Cartier Islands
   15. Australia
   16. Austria
   17. Azerbaijan
   18. The Bahamas
   19. Bahrain
   20. Bangladesh
   21. Barbados
   22. Bassas da India
   23. Belarus
   24. Belgium
   25. Belize
   26. Benin
   27. Bermuda
   28. Bhutan
   29. Bolivia
   30. Bosnia and Herzegovina
   31. Botswana
   32. Bouvet Island
   33. Brazil
   34. British Indian Ocean Territory
   35. British Virgin Islands
   36. Brunei
   37. Bulgaria
   38. Burkina Faso
   39. Burma
   40. Burundi
   41. Cambodia
   42. Cameroon
   43. Canada
   44. Cape Verde
   45. Cayman Islands
   46. Central African Republic
   47. Chad
   48. Chile
   49. China
   50. Christmas Island
   51. Clipperton Island
   52. Cocos (Keeling) Islands
   53. Colombia
   54. Comoros
   55. Congo, Democratic Republic of the
   56. Congo, Republic of the
   57. Cook Islands
   58. Coral Sea Islands
   59. Costa Rica
   60. Cote d'Ivoire
   61. Croatia
   62. Cuba
   63. Cyprus
   64. Czech Republic
   65. Denmark
   66. Dhekelia
   67. Djibouti
   68. Dominica
   69. Dominican Republic
   70. Ecuador
   71. Egypt
   72. El Salvador
   73. Equatorial Guinea
   74. Eritrea
   75. Estonia
   76. Ethiopia
   77. Europa Island
   78. Falkland Islands (Islas Malvinas)
   79. Faroe Islands
   80. Fiji
   81. Finland
   82. France
   83. French Guiana
   84. French Polynesia
   85. French Southern and Antarctic Lands
   86. Gabon
   87. Gambia, The
   88. Gaza Strip
   89. Georgia
   90. Germany
   91. Ghana
   92. Gibraltar
   93. Glorioso Islands
   94. Greece
   95. Greenland
   96. Grenada
   97. Guadeloupe
   98. Guam
   99. Guatemala
   100. Guernsey
   101. Guinea
   102. Guinea-Bissau
   103. Guyana
   104. Haiti
   105. Heard Island and McDonald Islands
   106. Holy See (Vatican City)
   107. Honduras
   108. Hong Kong
   109. Hungary
   110. Iceland
   111. India
   112. Indonesia
   113. Iran
   114. Iraq
   115. Ireland
   116. Isle of Man
   117. Israel
   118. Italy
   119. Jamaica
   120. Jan Mayen
   121. Japan
   122. Jersey
   123. Jordan
   124. Juan de Nova Island
   125. Kazakhstan
   126. Kenya
   127. Kiribati
   128. Korea, North
   129. Korea, South
   130. Kuwait
   131. Kyrgyzstan
   132. Laos
   133. Latvia
   134. Lebanon
   135. Lesotho
   136. Liberia
   137. Libya
   138. Liechtenstein
   139. Lithuania
   140. Luxembourg
   141. Macau
   142. Macedonia
   143. Madagascar
   144. Malawi
   145. Malaysia
   146. Maldives
   147. Mali
   148. Malta
   149. Marshall Islands
   150. Martinique
   151. Mauritania
   152. Mauritius
   153. Mayotte
   154. Mexico
   155. Micronesia, Federated States of
   156. Moldova
   157. Monaco
   158. Mongolia
   159. Montserrat
   160. Morocco
   161. Mozambique
   162. Namibia
   163. Nauru
   164. Navassa Island
   165. Nepal
   166. Netherlands
   167. Netherlands Antilles
   168. New Caledonia
   169. New Zealand
   170. Nicaragua
   171. Niger
   172. Nigeria
   173. Niue
   174. Norfolk Island
   175. Northern Mariana Islands
   176. Norway
   177. Oman
   178. Pakistan
   179. Palau
   180. Panama
   181. Papua New Guinea
   182. Paracel Islands
   183. Paraguay
   184. Peru
   185. Philippines
   186. Pitcairn Islands
   187. Poland
   188. Portugal
   189. Puerto Rico
   190. Qatar
   191. Reunion
   192. Romania
   193. Russia
   194. Rwanda
   195. Saint Helena
   196. Saint Kitts and Nevis
   197. Saint Lucia
   198. Saint Pierre and Miquelon
   199. Saint Vincent and the Grenadines
   200. Samoa
   201. San Marino
   202. Sao Tome and Principe
   203. Saudi Arabia
   204. Senegal
   205. Serbia and Montenegro
   206. Seychelles
   207. Sierra Leone
   208. Singapore
   209. Slovakia
   210. Slovenia
   211. Solomon Islands
   212. Somalia
   213. South Africa
   214. South Georgia and the South Sandwich Islands
   215. Spain
   216. Spratly Islands
   217. Sri Lanka
   218. Sudan
   219. Suriname
   220. Svalbard
   221. Swaziland
   222. Sweden
   223. Switzerland
   224. Syria
   225. Taiwan
   226. Tajikistan
   227. Tanzania
   228. Thailand
   229. Timor-Leste
   230. Togo
   231. Tokelau
   232. Tonga
   233. Trinidad and Tobago
   234. Tromelin Island
   235. Tunisia
   236. Turkey
   237. Turkmenistan
   238. Turks and Caicos Islands
   239. Tuvalu
   240. Uganda
   241. Ukraine
   242. United Arab Emirates
   243. United Kingdom
   244. United States
   245. Uruguay
   246. Uzbekistan
   247. Vanuatu
   248. Venezuela
   249. Vietnam
   250. Virgin Islands
   251. Wake Island
   252. Wallis and Futuna
   253. West Bank
   254. Western Sahara
   255. Yemen
   256. Zambia
   257. Zimbabwe
10. **Name of hospital/ institution:**
    1. Open-Ended Response
11. **Size of the population your CMR practice services (patient draw area):**
    1. < 100,000
    2. 100,000 - 250,000
    3. > 250,000 - 500,000
    4. > 500,000 - 1 million
    5. > 1 million
    6. I do not know
12. **Type of Institution where you primarily practice CMR (chose single best answer which describes the mission/purpose of the institution):**
    1. University/Academic/Teaching hospital
    2. Community/Non-academic hospital
    3. Government Institution (e.g. military, state-sponsored)
    4. Public assistance/Charity
    5. Research Institution
13. **Department responsible for the CMR scanner:**
    1. Cardiology
    2. Radiology/Imaging
    3. Shared between Radiology and Cardiology
    4. Other (please specify) Open-Ended Response
14. **Where is the primary location of your CMR program?**
    1. Both
    2. Hospital
    3. Imaging center/ outpatient facility
15. **Do you have a formal CMR training program with fellows in your Institution?**
    1. No
    2. Yes
    3. I do not know
16. **How long has your institution provided CMR as a service?**
    1. <1 year
    2. 1-2 years
    3. 3-5 years
    4. 6-10 years
    5. >10 years
    6. I do not know
17. **How many clinical CMR studies does your institution perform yearly?**
    1. < 100
    2. 101 - 300
    3. 301 - 500
    4. 501 - 999
    5. 1000 - 1999
    6. 2000 - 2999
    7. >/= 3000
    8. I do not know
18. **In comparison to last year how has your clinical CMR volume changed?**
    1. Increased 11-25%
    2. Increased 26-50%
    3. Increased >50%
    4. Remained the same (within +/- 10% change in volume)
    5. Decreased more than 10%
    6. I do not know
19. **How much time do you reserve for routine clinical CMR exams?**
    1. 30 minutes or less
    2. 31-45 minutes
    3. 46-60 minutes
    4. Greater than 60 minutes
20. **What % of your CMR studies are stress tests?**
    1. <10%
    2. 11-25%
    3. 26-50%
    4. >50%
    5. None, we do not do CMR stress
21. **Do you consider vascular MRA to be an important component to your CMR program?**
    1. No
    2. Yes

1. **Does low reimbursement for your professional time (reading fees) limit your availability to do CMR?**
   1. No
   2. Yes
   3. Not applicable
2. **Does the cost of CMR relative to other imaging modalities limit use of the technology?**
   1. No
   2. Yes
   3. I do not know
3. **Which vendors MR scanner do you use for CMR?**
   1. GE
   2. Philips
   3. Siemens
   4. Other (please specify) Open-Ended Response
4. **How much time do you reserve for your CMR slots?**
   1. 30 minutes or less
   2. 31-40 minutes
   3. 41-50 minutes
   4. 51-60 minutes
   5. >60 minutes
5. **Which are your primary indications for CMR (please choose best three)?**
   1. Myocarditis/Cardiomyopathies
   2. Suspected CAD/Ischemia in known CAD
   3. Myocardial viability
   4. Valvular heart disease
   5. Congenital heart disease
   6. Coronary vessels
   7. Other (please specify) Open-Ended Response
6. **You would benefit most from improvements in which area of cardiac MRI post-processing?**
   1. Function
   2. Strain Quantification
   3. Myocardial Delayed Enhancement
   4. T1/T2 Mapping
   5. 2D/4D Flow
   6. Stress Perfusion/Stress Function
7. **Will a 10-minute 4D flow acquisition ever REPLACE traditional CMR multi-slice function studies?**
   1. No, at best it will be used as an added test for a small percentage of patients
   2. No, but it will become an added test for a significant percentage of patients
   3. Yes, it will eventually replace a significant part of all current CMR function studies
8. **Will CMR strain imaging REPLACE traditional CMR multi-slice function studies in the next 5 years?**
   1. No way, multi-slice function does the job and is here to stay
   2. No, but CMR strain imaging will become an additional routine test
   3. Yes, methods based on in-plane myocardial tagging will replace multi-slice function
   4. Yes, single breathhold SENC-based imaging will replace multi-slice function
   5. Yes, speckle tracking methods will replace multi-slice function
9. **The main obstacle of cardiac MRI is:**
   1. High cost
   2. Limited accessibility
   3. Long scan time
   4. Scan setup (ECG prep)
   5. Technical difficulty
   6. Other (please specify) Open-Ended Response
10. **The clinical application that would be a game changer for cardiac MRI is:**
    1. Coronary imaging
    2. Electrophysiology
    3. Interventional MRI
    4. Metabolic imaging
    5. Valvular diseases
    6. Other (please specify) Open-Ended Response
11. **What are the main barriers to the growth of 4D flow at your institution? (rank top 3):**
    1. Competing technologies (echo, nuclear, PET)
    2. Turf battles
    3. Costs of 4D sequence
    4. Cost of 4D flow post-processing
    5. Lack of adequate reimbursement/Not covered by Third-party
    6. Access to scanners/Long waiting lists for studies
    7. Poor referring provider understanding of 4D flow application
    8. Long scan times/technical challenges of scanning
    9. Lack of clinical evidence to support use of 4D flow/lack of mention in clinical guidelines
    10. Lack of training opportunities for technologists to perform 4D flow
    11. Lack of training for physicians to perform/read 4D flow
12. **How is CMR viewed by your organization’s administration/leadership in relation to its importance to the organizations cardiovascular program?**
    1. Very important
    2. Somewhat important
    3. Neutral
    4. Not very important
    5. I do not know
13. **Is there broad awareness of the availability and indications for CMR at your institution?**
    1. No
    2. Yes
    3. Somewhat
    4. I do not know
14. **What imaging technologies at your institution do you feel compete the most against the use of CMR (rank top 2)?**
    1. Cardiac CT
    2. Echo
    3. Invasive angiography (cardiac cath)
    4. Nuclear PET
    5. Nuclear SPECT
    6. I do not know
15. **What imaging technologies at your institution do you feel compete the most against the use of CMR (rank top 2)?**
    1. Cardiac CT
    2. Echo
    3. Invasive angiography (cardiac cath)
    4. Nuclear PET
    5. Nuclear SPECT
    6. I do not know
16. **Are referring physicians at your Institution concerned about the use of gadolinium contrast agents in CMR/MRI imaging?**
    1. No
    2. Yes
    3. I do not know
17. **Which are the top referral indications for CMR at your Institution? (rank top 3)**
    1. Arrhythmia evaluation
    2. Assessment of viability
    3. Complex Congenial Heart Disease
    4. Evaluation of cardiomyopathy/CHF patients
    5. Evaluation of ischemic heart disease with stress testing
    6. Tissue characterization (amyloid, iron deposition, myocarditis)
    7. Complex Congenial Heart Disease
    8. Tissue characterization (amyloid, iron deposition, myocarditis)
    9. Evaluation of ischemic heart disease with stress testing
    10. Simple Congenital Heart Disease (ASD, VSD, PDA)
    11. I do not know
18. **Who are your top referring physicians? (top 3 referring)?**
    1. Adult/General Cardiologist (includes imagers)
    2. Adult/General Cardiologist (includes imagers)
    3. Cardiologist heart failure specialist
    4. Cardiovascular surgeon
    5. Electrophysiologist
    6. Emergency Medicine
    7. Internist/Family practitioner (Primary care)
    8. Interventional Cardiologist
    9. Pediatric/Adult congenital Cardiologist
    10. Other (Please specify below) Open-Ended Response
19. **What kind of training did you have (for CMR reading physicians only)?**
    1. 1 month at a specialized CMR Center
    2. 1-3 months at a specialized CMR Center
    3. 3-6 months at a specialized CMR Center
    4. 6-12 months at a specialized CMR Center
    5. More than 12 months at a specialized CMR Center
    6. Informal (self-taught/mini educational courses)
    7. Local training (in-house) with an experienced colleague
    8. Neither
20. **Do you think that the personnel involved in scanning the patients have enough physics and technical knowledge to overcome artifacts and technical related problems?**
    1. No
    2. Yes
    3. I don’t know
21. **Are you officially certified in CMR by:**
    1. SCMR
    2. EACVI
    3. Both SCMR and EACVI
    4. Other formal training
    5. Not applicable
22. **What kind of CMR training did the technologists/radiographers at your facility receive? (You may choose multiple responses)**
    1. Attended technologist’s track sessions at an SCMR meeting
    2. No formal training; I or another physician at my institution trained the technologist
    3. Other formal training
    4. I do not know
23. **What are the main barriers to the growth of CMR at your institution? (rank top 3)**
    1. Access to scanners/Long waiting lists for studies
    2. Competing technologies (echo, nuclear, PET)
    3. Costs of CMR study compared with other imaging modalities
    4. Lack of adequate reimbursement/Not covered by Third-party payment
    5. Long scan times/technical challenges of scanning
    6. Turf battles
    7. Lack of clinical evidence to support use of CMR/lack of mention in clinical guidelines
    8. Lack of training opportunities for technologists to perform CMR
    9. Poor referring provider understanding of CMR indications
    10. I do not know
    11. Other (Please specify below) Open-Ended Response
24. **Which of the following would you consider as a major technical limitation for CMR at your Institution (rank top 3)?**
    1. 4D flow
    2. Breath-hold duration
    3. EKG gating issues/arrhythmias
    4. Improved real-time imaging techniques
    5. Limited or no ability to scan patients with pacemakers/defibrillators
    6. Long scan times
    7. Non-contrast imaging techniques for MRA & perfusion
    8. Patient cooperation/claustrophobia
    9. Relaxation parameter (T1, T2, T2*) mapping
    10. Technologist limited expertise
    11. The need for additional hardware (i.e. Physiological monitoring, MR compatible infusion pumps, Anesthesia carts, etc)
    12. We have access to WIP sequences (compressed sense, realtime, single-shot), but gating remains the biggest challenge
    13. Departmental support
    14. None
    15. Other (Please specify below) Open-Ended Response
25. **What do you consider to be the next most promising application of MRI for clinical cardiovascular imaging (list top 2)?**
    1. 4D flow
    2. Compressed sensing
    3. Imaging techniques
    4. Improved real-time imaging techniques
    5. Non-contrast imaging techniques for MRA & perfusion
    6. Relaxation parameter (T1, T2, T2*) mapping
    7. None
    8. Other (Please specify below) Open-Ended Response
26. **How many scanners do you have available for CMR studies?**
    1. 1
    2. 2
    3. 3
    4. 4 or greater
27. **What field strength scanner(s) do you primarily use for clinical CMR?**
    1. 1.5 T
    2. 3 T
    3. 1.5 and 3 T
    4. > 3 T
    5. Animal scanners
28. **What brand(s) scanner do you primarily use for CMR studies? (check all that apply)**
    1. GE
    2. Philips
    3. Siemens
    4. Toshiba
29. **What software/workstation do you use primarily for clinical post-processing?**
    1. MRI vendor supplied software
    2. Third party software
    3. Other (please specify) What type (brand name)?
30. **Name:**
    1. Open-Ended Response
31. **Email:**
    1. Open-Ended Response

**DATA CURATION EXAMPLES:**

Several fields of the surveys were curated and normalized as follows: countries were normalized, i.e., some answers had “K” as the country of origin, while others had “Great Britain”; normalization integrated these records into one single tag “The United Kingdom"); the same curation process was performed for types of institutions and departments. In addition, concepts were simplified and grouped, which allowed us to present the data in 7 main groups: *demographics of the practitioner* (country of origin, gender, age range, primary practice type, profession and SCMR membership status); *training analysis* (level of CMR expertise, types of certifications and formal CMR training); staffing & service time (time reserved for CMR, considerations of MRA related to CMR, percentage of time dedicated to supervising/reading CMR and percentage of stress CMR tests); *institutional analysis* (type of Institution, department responsible for CMR, primary location of CMR program, years of CMR service, service area size, quantity of clinical CMR studies, percentage of total time designated to CMR, brands of CMR equipment, image post-processing software and third party software used); *growth and barriers* (top referring physicians, competing technologies, main barriers in the respondents’ institution and top referral indications for CMR); *technical questions* (most beneficial improvements in CMR, main obstacle of CMR, next promising application of MRI for clinical cardiovascular imaging, 4D flow vs. traditional CMR, game changers and CMR strain imaging vs. traditional CMR); and finally, the *cost structure* (CMR vs. transthoracic echo, CMR vs. transesophageal echo, CMR vs. dobutamine echo, CMR vs. SPECT, CMR (viability) vs. Nuclear (SPECT), CMR (viability) vs. Nuclear (PET), CMR vs. Nuclear (PET), tissue characterization and CMR vs. other imaging modalities).

**Figures and Tables:**

**Figure 1S**. World maps (**Figure 1S.1** Top five respondent countries in the survey, **Figure 1S.2** Responders from North, Central, and South America, **Figure 1S.3** from Europe, **Figure 1S.4** from the Middle East, **Figure 1S.5** from Asia, **Figure 1S.6** from Africa, **Figure 1S.7** from Oceania). Color coding only indicates different regions.

**Table 1S.** SCMR membership distribution

**Table 2S.** Respondents’ background

**Table 3S**. CMR logistics

**Table 4S.** CMR indications and referrals

**Table 5S.** CMR main barriers

**Figure 2S.** CMR main referring physicians segregated by High vs. Low volume center analysis (<1000 vs. >1000 CMR studies per year).

**Figure 3S.** Community size of CMR service area segregated by High vs. Low volume center analysis (<1000 vs. >1000 CMR studies per year).

## **Figure 1S.1Top five respondent countries in the survey**


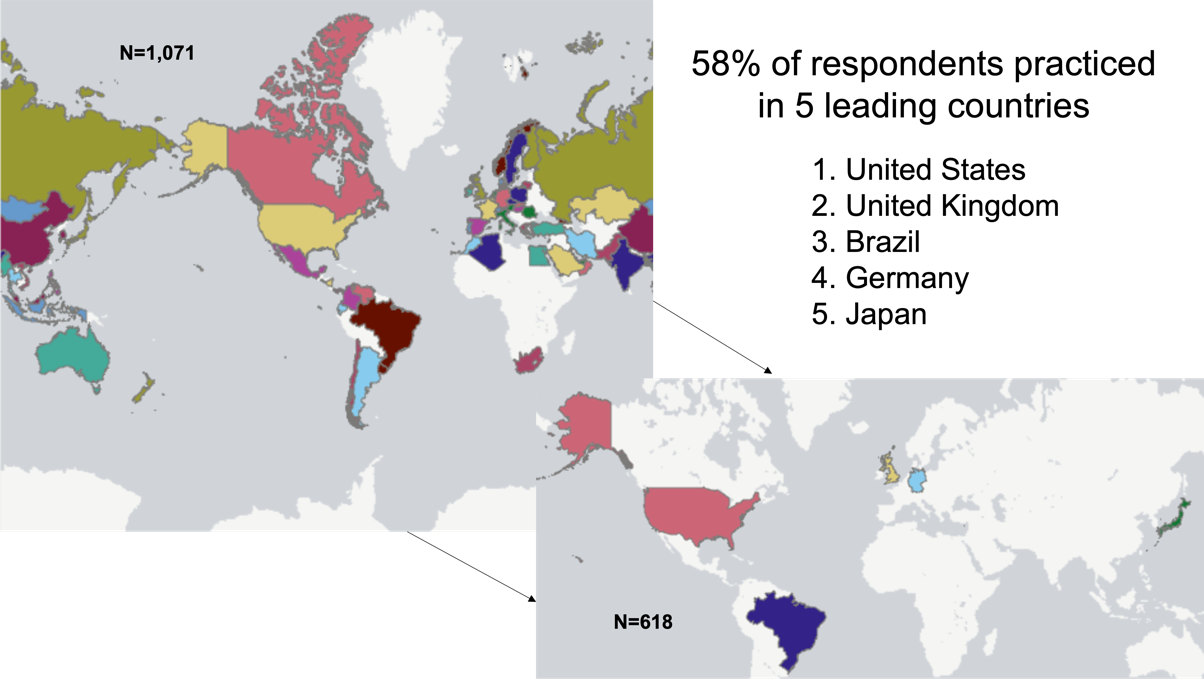


## **Figure 1S.2 Respondent countries from North, Central, and South America**


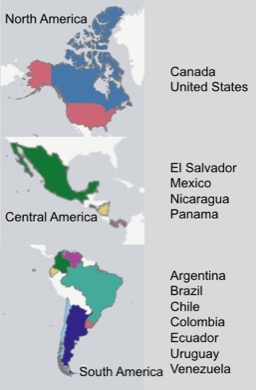


## **Figure 1S.3 Respondent countries from Europe**


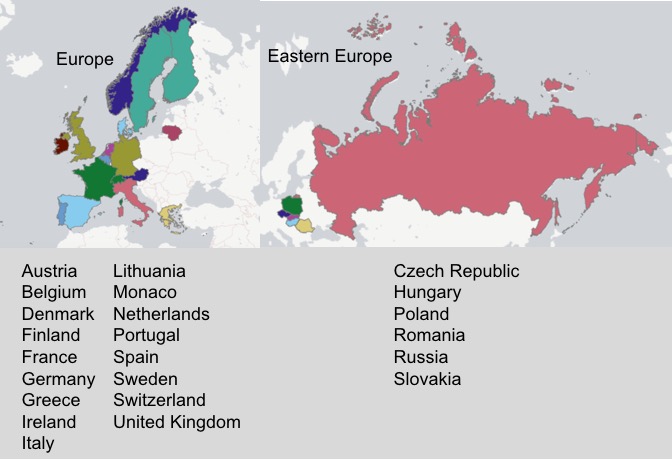


## **Figure 1S.4 Respondent countries from the Middle East**


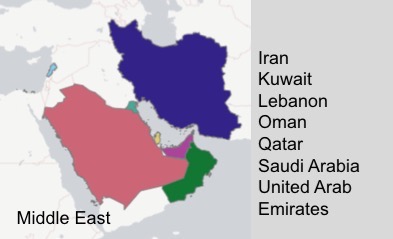


## **Figure 1S. 5 Respondent countries from Asia**


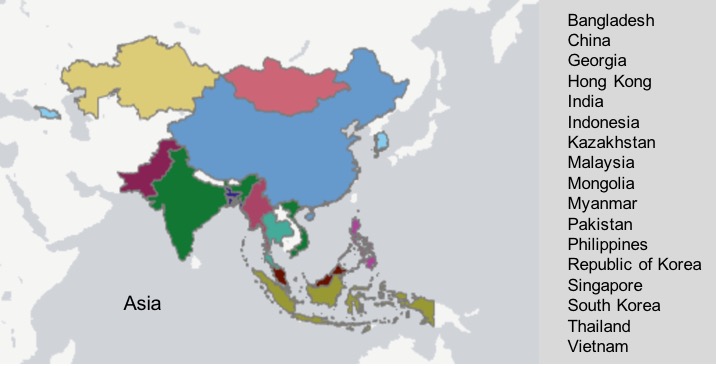


## **Figure 1S. 6 Respondent countries from Africa**


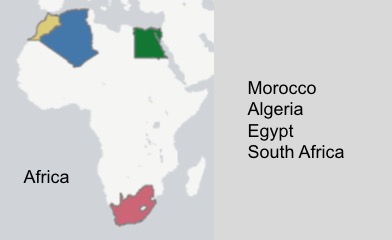


## **Figure 1S. 7 Respondent countries from Oceania**


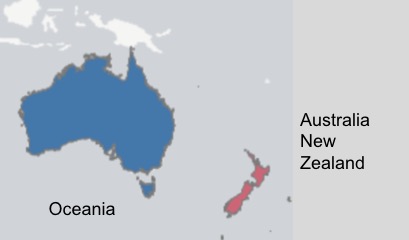


**Table 1S. SCMR membership distribution**

| **SCMR Membership** | | |
| --- | --- | --- |
| **Membership** | **n=1,090** | **%** |
| Member | 564 | 52 |
| Non-Member | 526 | 48 |
| **Type of membership** | **n=564** | **%** |
| Full | 363 | 64 |
| Associate | 69 | 12 |
| Trainee | 96 | 17 |
| Technologist | 36 | 6 |

**Table 2S. Respondents background**

| **Respondent’s background** | | |
| --- | --- | --- |
| **Background** | **n=884** | **%** |
| Adult cardiologist | 423 | 48 |
| Adult radiologist | 193 | 22 |
| Pediatric cardiologist | 96 | 11 |
| Pediatric radiologist | 56 | 6 |
| Technologist | 50 | 6 |
| Scientist-non clinical | 30 | 3 |
| Medical industry | 21 | 2 |
| Other | 9 | 1 |
| Administration | 6 | 1 |

**Table 3S. CMR logistics**

| **CMR logistics** | | |
| --- | --- | --- |
| **Time reserved for CMR studies (minutes)** | **n=654** | **%** |
| >60 | 118 | 18 |
| 46-60 | 342 | 52 |
| 31-45 | 154 | 24 |
| <30 | 40 | 6 |
| **Professional time used for supervising/reading CMR (%)** | **n=564** | **%** |
| <10 | 146 | 22 |
| 11-25 | 199 | 30 |
| 26-50 | 177 | 27 |
| 51-75 | 76 | 11 |
| >75 | 64 | 10 |

**Table 4S. CMR indications and referrals**

| **CMR indications and referrals** | | |
| --- | --- | --- |
| **Main indications** | **n=585** | **%** |
| Evaluation of cardiomyopathy | 213 | 36 |
| Viability assessment | 134 | 23 |
| Evaluation of ischemic heart disease | 97 | 17 |
| Complex congenital heart disease | 54 | 9 |
| Tissue characterization | 43 | 7 |
| Arrhythmia evaluation | 29 | 5 |
| Vascular (MRA) | 6 | 1 |
| ASD, VSD, PDA evaluation | 5 | 1 |
| Valvular heart disease | 4 | 1 |
| **Main referring physicians** | **n=818** | **%** |
| Adult/general cardiologist (including imagers) | 435 | 53 |
| Adult/general cardiologist (not imagers) | 245 | 30 |
| Pediatric/adult congenital cardiologist | 58 | 7 |
| Cardiologist heart failure specialist | 37 | 5 |
| Electrophysiologist | 20 | 2 |
| Interventional cardiologist | 17 | 2 |
| Internist/family practitioners (primary care) | 4 | 0.5 |
| CV surgeon | 2 | 0.2 |

**Table 5S. CMR main barriers**

| **CMR main barriers** | | |
| --- | --- | --- |
| **Main barriers** | **n=556** | **%** |
| Access to scanners | 145 | 26 |
| High cost | 132 | 24 |
| Competing technologies | 110 | 20 |
| Lack of training | 79 | 14 |
| Long scan times | 51 | 9 |
| Internal conflicts | 39 | 7 |

**
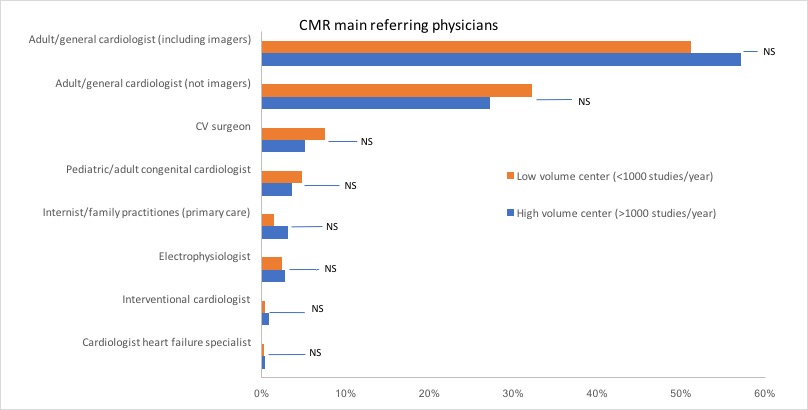
Figure 2S. High vs. Low volume center analysis of main CMR referring physicians (<1000 vs. >1000 CMR studies per year)**

**Figure 3S. Community size of the CMR area of service**

**
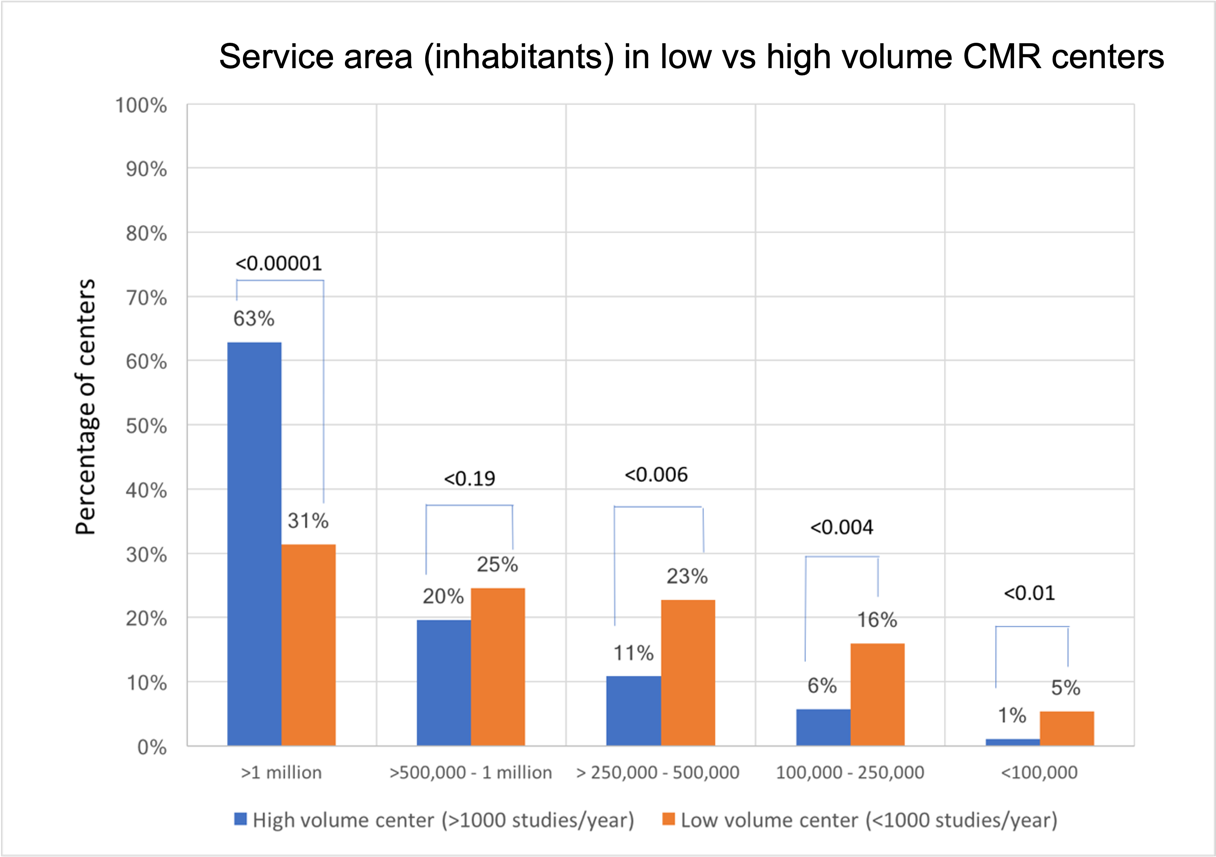
**

**FIGURE AND TABLE LEGENDS:**

**Figure 1S.** World maps. **Figure 1S.1 Top** five respondent countries in the survey. This figure shows the world map of respondent counties, highlighting the top 5 respondents that achieved 58% survey’s total responders. **Figure 1S.2.** Respondent countries from North, Central, and South America, **Figure 1S.3.** Respondent countries from Europe, **Figure 1S.4.** Respondent countries from the Middle East, **Figure 1S.5.** Respondent countries from Asia, **Figure 1S.6.** Respondent countries from Africa, **Figure 1S.7.** Respondent countries from Oceania.

**Table 1S.** SCMR Membership Distribution. This table shows SCMR membership and type distribution among total responders of the survey. The sample size of each category is different since not all responders answered all questions.

**Table 2S.**  Respondents’ Background. This table shows professional background among the total responders of the survey.

**Table 3S.** CMR logistics. This table shows two aspects of CMR logistics, the time reserved for CMR studies at the scanner in minutes and the professional time used for supervising/reading CMR studies in the percentage of total professional time. The sample size of each category is different since not all respondents answered all questions.

**Table 4S.** CMR indications and referrals. This table shows CMR's main indications and referring physicians. The sample size of each category is different since not all respondents answered all questions. **Abbreviations: ASD:** atrial septal defect, **VSD:** ventricular septal defect, **PDA:** patent ductus arterioles, **MRA:** magnetic resonance angiography, **CV:** cardiovascular.

**Table 5S.** CMR main barriers. This table shows the top reported barriers to CMR program growth.

**Figure 2S.** High vs. Low volume center analysis of main CMR referring physicians (<1000 vs. >1000 CMR studies per year). This figure shows the CMR main referring physicians when the respondent centers are divided into two large groups; low volume centers (those with a volume of <1000 CMR studies per year of any kind), and those considered of large volume centers (with a volume of >1000 CMR studies per year of any kind). **Abbreviations: CMR:** cardiovascular magnetic resonance. **NS:** non-significant, **CV:** cardiovascular.

**Figure 3S.** Community size of the CMR area of service. This figure shows the service area size of the CMR site according to the approximate number of inhabitants in range when the respondent centers are divided into two large groups; low volume centers (those with a volume of <1000 CMR studies per year of any kind), and those considered of large volume centers (with a volume of >1000 CMR studies per year of any kind). **Abbreviations: CMR:** cardiovascular magnetic resonance.
